# Hypermethylation and down-regulation of *DLEU2* in paediatric acute myeloid leukaemia independent of embedded tumour suppressor *miR-15a/16-1*

**DOI:** 10.1186/1476-4598-13-123

**Published:** 2014-05-24

**Authors:** Leah Morenos, Zac Chatterton, Jane L Ng, Minhee S Halemba, Mandy Parkinson-Bates, Francoise Mechinaud, Ngaire Elwood, Richard Saffery, Nicholas C Wong

**Affiliations:** 1 Cancer & Disease Epigenetics, Murdoch Childrens Research Institute, Melbourne, Victoria, Australia; 2 Department of Paediatrics, The University of Melbourne, Melbourne, Victoria, Australia; 3 Cancer Research, Murdoch Childrens Research Institute, Melbourne, Victoria, Australia; 4 BMDI Cord Blood Bank, Murdoch Childrens Research Institute, Melbourne, Victoria, Australia; 5 Current address: Ludwig Institute of Cancer Research, Olivia Newton John Cancer and Wellness Centre, Austin Hospital, Heidelberg, Victoria, Australia

**Keywords:** MicroRNA, DNA ethylation, Leukaemia, Paediatric, AML, *DLEU2*, *miR-15a/16-1*, Cytogenetics

## Abstract

**Background:**

Acute Myeloid Leukaemia (AML) is a highly heterogeneous disease. Studies in adult AML have identified epigenetic changes, specifically DNA methylation, associated with leukaemia subtype, age of onset and patient survival which highlights this heterogeneity. However, only limited DNA methylation studies have elucidated any associations in paediatric AML.

**Methods:**

We interrogated DNA methylation on a cohort of paediatric AML FAB subtype M5 patients using the Illumina HumanMethylation450 (HM450) BeadChip, identifying a number of target genes with p <0.01 and Δβ >0.4 between leukaemic and matched remission (n = 20 primary leukaemic, n = 13 matched remission). Amongst those genes identified, we interrogate *DLEU2* methylation using locus-specific SEQUENOM MassARRAY® EpiTYPER® and an increased validation cohort (n = 28 primary leukaemic, n = 14 matched remission, n = 17 additional non-leukaemic and cell lines). Following methylation analysis, expression studies were undertaken utilising the same patient samples for singleplex TaqMan gene and miRNA assays and relative expression comparisons.

**Results:**

We identified differential DNA methylation at the *DLEU2* locus*,* encompassing the tumour suppressor microRNA *miR-15a/16-1* cluster. A number of HM450 probes spanning the *DLEU2/Alt1* Transcriptional Start Site showed increased levels of methylation in leukaemia (average over all probes >60%) compared to disease-free haematopoietic cells and patient remission samples (<24%) (p < 0.001). Interestingly, DLEU2 mRNA down-regulation in leukaemic patients (p < 0.05) was independent of the embedded mature miR-15a/16-1 expression. To assess prognostic significance of *DLEU2* DNA methylation, we stratified paediatric AML patients by their methylation status. A subset of patients recorded methylation values for *DLEU2* akin to non-leukaemic specimens, specifically patients with sole trisomy 8 and/or chromosome 11 abnormalities. These patients also showed similar miR-15a/16-1 expression to non-leukaemic samples, and potential improved disease prognosis.

**Conclusions:**

The *DLEU2* locus and embedded miRNA cluster *miR-15a/16-1* is commonly deleted in adult cancers and shown to induce leukaemogenesis, however in paediatric AML we found the region to be transcriptionally repressed. In combination, our data highlights the utility of interrogating DNA methylation and microRNA in combination with underlying genetic status to provide novel insights into AML biology.

## Background

Acute myeloid leukaemia (AML) is the third most common form of leukaemia in children, typically characterised by the rapid proliferation of primitive haematopoietic myeloid progenitor cells [1]. Paediatric AML is a highly heterogeneous disease, which presents a major barrier towards the development of accurate disease classification, risk stratification and targeted therapies within the clinic. The French-American-British (FAB) [2] and more recently World Health Organisation (WHO) [3] classifications of leukaemia take into account cell morphology, cytogenetic aberrations and common genetic lesions. However, not all patients fall into these well-defined categories. Additionally, the recurrent chromosomal and genetic lesions frequently found in AML fail to induce leukaemogenesis and do not explain the recognised clinical heterogeneity [4,5].

One of the hallmarks of nearly all human cancers is the disruption of the epigenetic profile, including gross aberrations in DNA methylation. Increasing evidence in adult AML has indicated that epigenetic events play critical roles in the onset, progression, and outcome of AML [6] and may help tailor disease treatment. However, the need for similar elucidations in childhood disease is paramount. Aberrant methylation of cytosine residues at palindromic CpG sites (often clustered in dense CpG ‘islands’) near gene promoter regions is widely studied in carcinogenesis and haematological malignancies [6,7]. It is now well established that elevated DNA methylation is an important mechanism of gene transcriptional inactivation [8,9] and genes such as *ESR1*, *IGSF4* and *CDKN2B/p15* are epigenetically silenced in adult leukaemia [6]. Previous studies have subdivided adult AML into 16 epigenetic sub-groups based on DNA methylation signatures, correlating with patient clinical outcome and distinct from both normal haematopoietic cells and normal stages of myeloid differentiation [4]. Despite such emerging findings in an adult context, the utility of individual DNA methylation disruptions in paediatric AML has yet to be fully evaluated [6].

MicroRNA (miRNA) represent an alternative epigenetic regulator, having been implicated in the regulation of critical gene expression networks in plants and animals. The role of miRNA in haematopoiesis, cancer and disease is also beginning to be appreciated [10,11]. The global influence of individual miRNA on the genome is difficult to dissect, as miRNA can modulate the expression of hundreds of genes, and each gene can harbour binding sites for several miRNA [12]. Human miRNA are initially transcribed (pri-miRNA), and processed by several complexes to form a 70 bp hairpin-loop (pre-miRNA) [13]. After successive enzymatic steps, a miRNA:miRNA* complementary duplex is formed where the ‘functional’ strand is combined with RISC (RNA Induced Silencing Complex) and Argonaute proteins to guide, and inhibit, specific target messenger RNA (mRNA) through base pair recognition [14,15]. However, the miRNA transcriptome is becoming increasingly complex, emphasised by Next Generation Sequencing (NGS) technologies. NGS has highlighted that alternate miRNA* transcripts, as well as miRNA sequence variants (isomiRs [16]) may play a biological role, similar to their canonical miRNA relatives [17,18].

Links between miRNA deregulation and cancer diagnosis were first identified in adult Chronic Lymphoblastic Leukaemia (CLL), where the loss or down-regulation of tumour-suppressing miRNA cluster *miR-15a/16-1* directly caused leukaemic transformation [19,20]. At present, no such association has been identified for childhood leukaemia. The expression of paediatric disease-associated miRNA has to date only identified a distinction between leukaemia of different lineages and the differentiation of rearranged AMLs within a limited number of cytogenetic subtypes [21,22]. Paediatric *MLL* can be distinguished from others by differentially expressed miR-126, miR-146a, miR-181a/b/d, miR-100, miR-21, miR-196a/b, miR-29 and miR-125b [21]. However concordance among studies is often low and the mechanism of deregulation is often unknown [22,23].

Genes encoding miRNA can be regulated epigenetically in a similar manner to protein coding genes [22]. Studies have demonstrated epigenetically regulated miRNA in adult AML, including hypermethylation and down-regulation of miR-124a and associated deregulation of target mRNA *EVI1, CEBPA* and *CDK6* independent of diagnostic cytogenetic subtype (reviewed in [22]). Additionally, miR-193a targeting *KIT*, and miR-14b targeting *CREB* have been identified in adult investigations as specifically controlled by DNA methylation (reviewed in [22]). However, the identification of DNA methylation and miRNA expression connections in paediatric leukaemia is lacking.

Paediatric AML has distinct cytogenetic and clinical features relative to their adult counterparts [5,21,24-26]. Therefore, there is a critical need to improve our understanding of the biology of childhood leukaemia as separate entities, distinct from adult disease. Cognisant of this, we aimed to identify differential DNA methylation within paediatric AML on a genome-scale using defined clinical subtypes and age-matched controls. We identified a number of significantly altered DNA methylation loci, with associated gene and miRNA expression change, between paediatric AML and non-leukaemic counterparts. Specifically we describe here the epigenetic deregulation of *DLEU2*, which has associated alterations in downstream *miR-15a/16-1* miRNA cluster expression.

## Results and discussion

### The *DLEU2* gene is specifically hypermethylated and repressed in paediatric AML subtype M5

The FAB subtype M5 (monocytic/blastic leukaemia) is a distinct subtype with characteristic chromosomal abnormalities including t(8; 16), +8 and various translocations involving 11q23 and the *MLL* locus such as t(9;11), t(10;11), t(11;19) and others [27]. AML subtype M5 also has a high proportion of cytogenetically normal (CN-AML) patients [27], and those with complex karyotypes [28]. A combination of these factors add to the overall unfavourable outcome of paediatric M5 diagnosis [29]. Genome-scale methylation profiling of AML M5 bone marrow samples identified 3,352 significantly differentially methylated probes (DMPs) between paediatric AML FAB M5 (n = 20) and matching non-leukaemic (n = 17) samples. Applying more stringent feature selection criteria of an adjusted p-value <0.01 and ∆β of >0.4 reduced the number of DMPs to 137 (Additional file 1).

**Figure 1:**
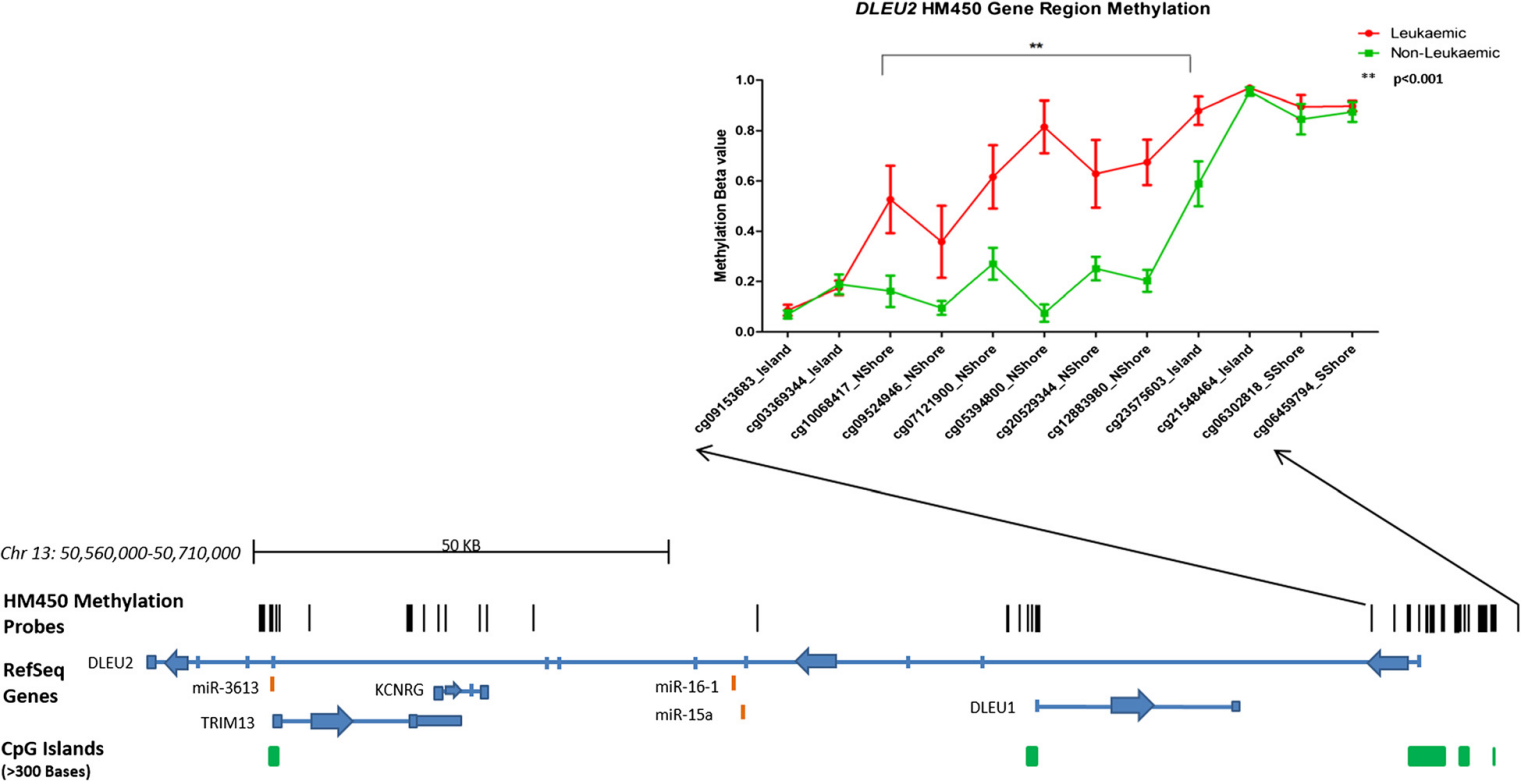
**Regional interrogation of the *DLEU2 *gene including significantly differentially methylated probes identified by HM450 analysis. *Bottom****:* Distribution of HM450 methylation probes across the *DLEU2* region of 13q4. Genes located in this region include: *DLEU1, DLEU2, miR15a/16-1* microRNA cluster, *TRIM13, KCNRG* and *miR-3613.****Top****:* HM450 probes identified as significantly differentially methylated between paediatric AML (FAB subtype M5) and non-leukaemic specimens have been plotted against genomic location. The leukaemic group (n = 16) refers to diagnostic bone marrow from paediatric patients. Non-leukaemic group (n = 11) consists of CD sorted cell populations (CD19+, CD33+, CD34+, CD45+) and patient remission specimens. Differential methylation is concentrated in the promoter region of the *DLEU2/Alt1* long transcript, which also falls into the body region of *DLEU1* within 3 CpG islands (Chr 13: 50, 690,000-50,708,000). Mean methylation β values and 95% CI are shown. Data for leukaemic samples are red and non-leukaemic samples in green. Methylation values range from 0 (0%, no detected methylation) to 1.0 (100% fully methylated). Significantly differentially methylated regions between leukaemic and non-leukaemic samples to p < 0.001 are highlighted with **.

The list of DMPs included several localising to the long non-coding RNA *DLEU2* and the embedded miRNA cluster *miR-15a/16-1*[19,20], previously implicated in adult leukaemic [19,20,30]. To date, disruption of this region has not been observed in paediatric cancers and as such we chose to focus on *DLEU2* in subsequent analysis. A total of three *DLEU2* DMPs had an adjusted p-value <0.01 and ∆β of >0.4 (cg05394800, cg20529344, cg12883980). These probes were located within three CpG islands (Chr 13: 50, 690,000-50,708,000: UCSC human hg19 assembly) at the *DLEU2/Alt1* transcriptional start site (TSS) and ‘north shore’ (Figure 1), a region up to 2 kb upstream from the *DLEU2/Alt1* TSS CpG island under investigation [31]. Henceforth this will be referred to as the *DLEU2* promoter.

A divergent DNA methylation profile was observed between all cases of paediatric AML with elevated methylation (mean 64% (48-80% CI) across significant probes and non-leukaemic samples (23% methylation; 7-39% CI of mean) (p < 0.001 for all significant probes) (Figure 1; Additional file 2). DNA methylation levels at CpG sites interrogated by cg05394800, cg20529344, cg12883980 were confirmed using SEQUENOM EpiTYPER MassArray (Additional file 3 and Additional file 4) in the discovery sample set of 20 leukaemic patients and 17 non-leukaemic controls, as well as a validation set of paediatric AML M5 patients with heterogeneous cytogenetic diagnoses (n = 19) (CN-AML (n = 5), *MLL* (n = 7), *RUNX1* (n = 2), *WT1* (n = 1)) (Additional file 5). This analysis confirmed specific *DLEU2* promoter hypermethylation in paediatric AML patients (p < 0.001) (Additional file 4), with additional genes in this region, including *DLEU1*, *hsa-miR-15a/16-1* microRNA cluster, *TRIM13*, *KCNRG* and *hsa-miR-3613* showing no differences in methylation between cases and controls (Additional file 6). This highlights the potential regional specificity of the observed *DLEU2* methylation change in association with AML.

**Figure 2:**
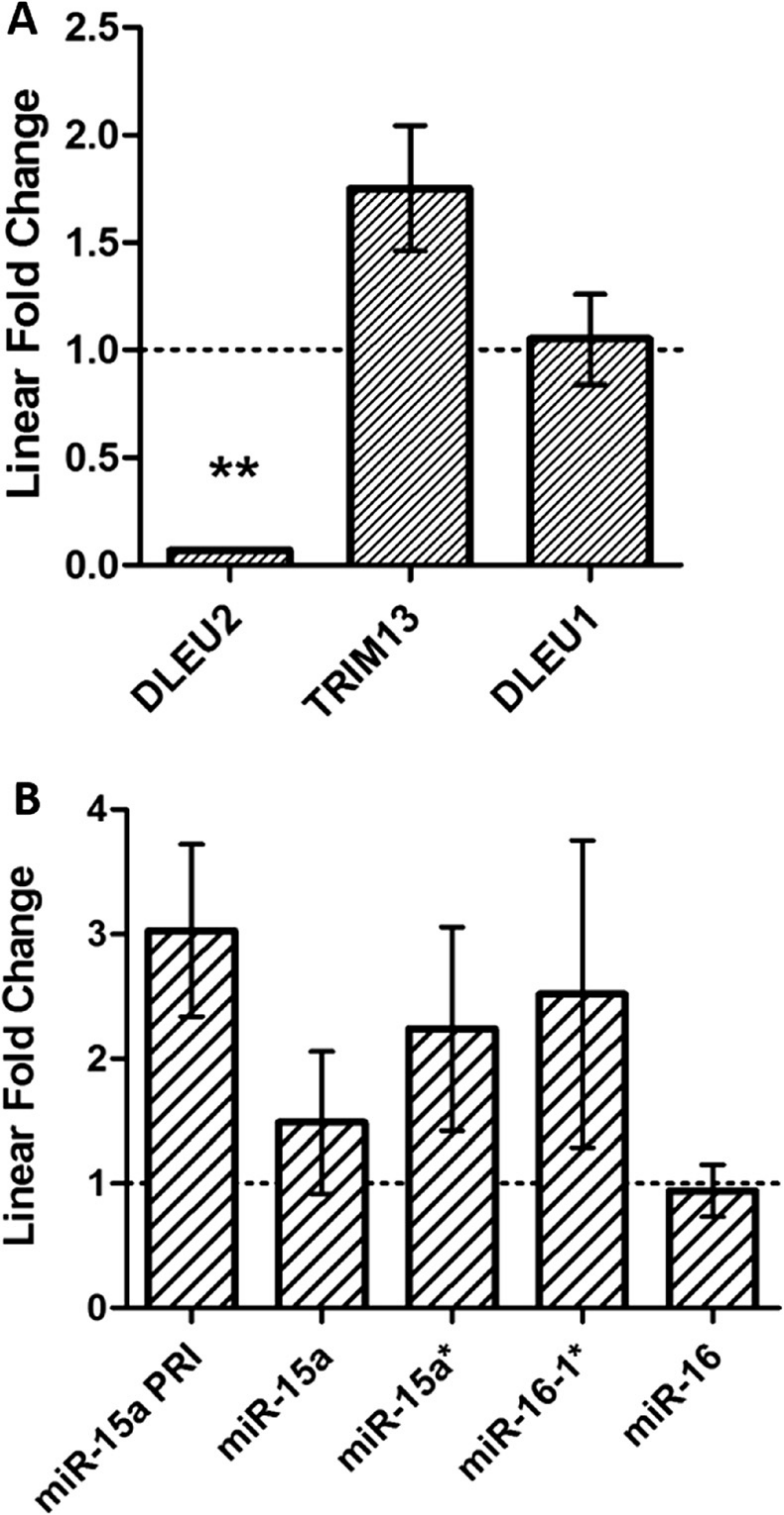
**Expression analysis of DLEU2 and miR-15/16 in paediatric AML.** Here we interrogate the gene, miRNA and precursor miRNA expression in paediatric AML compared to non-leukaemic. This interrogation includes *DLEU2* and embedded *miR-15a/16-1* on chromosome 13q4. The leukaemic group refers to diagnostic bone marrow from paediatric patients. The non-leukaemic group consists of CD sorted cell populations (CD19+, CD33+, CD34+, CD45+) and patient remission specimens, and is represented by the dashed line at Y = 1. Fold Change (FC) is plotted using normalized data and the 2^-ΔΔCt^ method ± SD, and shows the fold change calculated from the means of each group. **A**. Gene expression including *DLEU1*, *DLEU2* and *TRIM13* in paediatric AML (n = 10) compared to non-leukaemic (n = 13) expression. *DLEU2* shows a significant down-regulation in AML compared to non-leukaemic expression (0.07 FC, p = 0.014 represented by **), however there is no significant change in expression for *TRIM13* or *DLEU1*. **B**. Mature microRNA expression, including primary precursor transcript (PRI) and alternate miRNA expression (*), from the *miR-15a/16-1* miRNA cluster embedded within *DLEU2* for paediatric AML (n = 28, including the 10 used in Figure 2A) compared to non-leukaemic specimens (n = 30, including the 13 used in Figure 2A). Here the miR-15a/16-1 PRI transcript is 3.03-fold higher in expression compared to non-leukaemic expression. Additionally, miR-16-1* (2.52 FC), miR-15a* (2.24 FC) and miR-15a (1.5 FC) also show increases in expression in paediatric AML. No significant change in expression was observed for miR-16 (0.94 FC) in paediatric AML compared to non-leukaemic expression.

We observed a significant down-regulation of DLEU2 gene expression in paediatric AML (0.07 Fold Change (FC); p = 0.014), and a significant inverse correlation between promoter DNA methylation and gene expression levels (p = 0.0001, Additional file 7). Recent studies in adult CLL have also identified a negative correlation between *DLEU2* promoter methylation (*DLEU2/Alt1)* and gene expression [32]. Interestingly there was no change in gene expression for any other genes in this region (TRIM13 = 1.75 FC; DLEU1 = 1.05 FC. Figure 2A).

### *DLEU2* and embedded *miR-15a/16-1* are regulated independently in paediatric AML

The *miR-15a/16-1* cluster has been described to have potent tumour suppressor activity, targeting numerous oncogenic and cell cycle regulatory genes [19,33]. The cluster is embedded within intron 4 of *DLEU2*, and has been speculated that expression is driven by the *DLEU2* promoter [20,30]. However, we found no correlation between DLEU2 expression and miR-15a/16-1 expression in paediatric AML, nor down-regulation of the *miR-15a/16-1* miRNA cluster in relation to increasing *DLEU2* promoter DNA methylation (Figure 2B). In contrast to previous reports for adult leukaemia, no significant change in mature miR-16 expression was observed between paediatric AML and control samples, a result we have reported elsewhere [34]. These observations are independent of the homologous miR-16 cluster embedded in *SMC4* on chromosome 3q26, which shows no significant expression or DNA methylation changes in association with AML (Additional file 8). Similar results have been reported for adult CLL [32]. Taken together this data suggests an alternate mode of regulation for the *miR-15a/16-1* cluster, outside of the *DLEU2* promoter region.

Interestingly, recent research has indicated the processing mechanisms of miRNA may be affected by cancer, such that mature miRNA expression becomes disassociated from precursor miRNA levels, and also from the levels of the host gene [35]. We found the *miR-15a/16-1* primary precursor transcript is in fact expressed up to three-fold higher in AML patients compared to non-leukaemic counterparts (Figure 2B). The increase in primary transcript appears to correspond to an increase in mature miR-16-1* (2.52 FC) and miR-15a* (2.2 FC) expression, and a moderate increase in miR-15a (1.5 FC). However, individual patient samples show differential degrees of over-expression of one, or all, of the mature species despite the common precursor (Additional file 9).

Canonical *miR-15a/16-1* microRNA species target numerous oncogenic and integral cell cycle regulatory genes such as *BCL-2, MCL-1, CCND1, CDK6, BMI-1, RASSF5, IGSF4, c-MYB* and *WNT3A*[33,36-42]. To identify the potential significance of increasing alternate *miR-15a/16-1* transcripts over their canonical counterparts in paediatric AML, we investigated miRNA target genes using prediction tools, and undertook gene ontology analysis. We found miR-16-1* is three-fold enriched for targeting components of the RNA processing and splicing machinery such as hnRNP regulators, *SRRM1* and *NUDT2.* Down-regulation of these genes by increased expression of a targeting miRNA may be contributing to miRNA/host gene disassociation in *DLEU2/miRNA-15a/16-1* expression (Additional file 10)*.* One target of miR-15a* was found to be *ADORA2A,* an important G-protein receptor critical in tissue-specific and systemic inflammatory responses [43,44]. We have identified the down-regulation of ADORA2A expression within paediatric AML, not mediated through DNA methylation, but potentially through the up-regulation of the miR-15a/16-1 cluster (data not shown). Moreover, miR-15a* and miR-16-1* are 60–90 fold enriched for targeting genes involved in the intrinsic apoptotic pathway such as *BCL-2 L11*, *PPIF* and *DNM1L* (Additional file 10). The inhibition of these genes to act upon the cell, mediated through targeting miRNA, can potentially encourage continued cell growth and perpetuation of AML, through negating apoptotic signalling. The over expression of alternate mature miRNA in paediatric AML may also have consequences for integral cell cycle pathways, but through different mechanisms to their canonical counterparts.

### *DLEU2* interrogation identifies a novel subclass of paediatric AML

In addition to assessing the association of *DLEU2* promoter DNA methylation and *miR-15a/16-1* miRNA expression with paediatric AML, we also assessed correlations with distinct clinical and diagnostic variables. We found no significant association between *DLEU2* methylation/expression with sex, age of disease onset or relapse status nor common gene abnormalities such as *FLT3* or *MLL*, as has been found in a number of other association studies [6,45]. *DLEU2* methylation clustering based on diagnostic FAB subtype revealed a wide range of values (M5a: 16-91%; M5b: 48-90%; ‘Other’ subtypes: 51-93%. Additional file 11), indicating mean methylation in isolation does not stratify paediatric AML subtype. Additionally, we found there to be no correlation between percentage of leukaemic blasts at patient diagnosis and the DNA methylation status at the *DLEU2* promoter region (data not shown).

**Figure 3:**
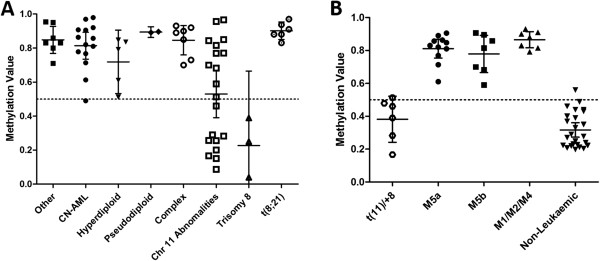
**Interrogation of paediatric AML by clinically defined cytogenetic and FAB subtypes alongside *DLEU2 *promoter methylation.** Paediatric AML diagnostic cytogenetic and subtyping analyses were specifically investigated, including those with known gene abnormalities and CN-AML cases. We investigate here the DNA Methylation of *DLEU2/Alt1* promoter region to 95% CI. DNA methylation values range from 0.0 (0% no detected methylation) to 1.0 (100% fully methylated). The leukaemic group refers to diagnostic bone marrow from paediatric patients. Non-leukaemic group consists of CD sorted cell populations (CD19+, CD33+, CD34+, CD45+) and patient remission specimens. **A**. DNA methylation for *DLEU2* HM450 probes (cg12883980, cg20529344, cg5394800) according to cytogenetic type, showing heterogeneous DNA methylation outcomes. Of note, a subset of patients with observable chromosome 11 and trisomy 8 abnormalities have a reduced DNA methylation compared to all other AML abnormalities. **B**. A subset of *DLEU2* DNA methylation results for paediatric AML chromosome 11 and trisomy 8 abnormality patients from Figure 3A fall into a range akin to non-leukaemic specimens. Grouping these patients, regardless of clinical subtyping (M5a, M5b or M1/M2/M4), forms an additional sub-group, denoted here as ‘t(11)/+8’. The DNA methylation values obtained for probe cg12883980 are depicted, as an indication of the methylation at the *DLEU2* promoter. t(11)/+8 are not differentially methylated compared to non-leukaemic specimens (t(11)/+8 sub-group Mean Methylation (MM) = 0.38, non-leukaemic MM = 0.32. p = 0.22). M5a, M5b and M1/M2/M4 AMLs (with the removal of t(11)/+8 patients) have become increasingly hypermethylated compared to non-leukaemic methylation (M5a MM = 0.81, M5b MM = 0.78, M1/M2/M4 MM = 0.87. p < 0.0001), and significantly different from t(11)/+8 (M5a/b p < 0.05; M1/M2/M4 p < 0.0001).

Previous adult leukaemia studies have identified novel methylation subgroups, independent of cytogenetic profile [4,46]. We identified a subset of patients displaying low *DLEU2* promoter methylation comprising patients with trisomy 8 and/or chromosome 11q abnormalities, independent of *MLL* rearrangements (separated into a t(11)/+8 subgroup - Figure 3A, Additional file 11). This delineation identified t(11)/+8 subgroup as having comparable *DLEU2* methylation levels to non-leukaemic specimens (Figure 3B), and significantly lower methylation compared to the remaining paediatric AML samples (M5a/b p < 0.05; M1/M2/M4 p < 0.0001 Figure 3B). Classifying t(11)/+8 patients as a separate subgroup further delineated M5a, M5b and M1/M2/M4 AML patients from non-leukaemic counterparts according to average methylation status (M5a MM = 0.81, M5b MM = 0.78, M1/M2/M4 MM = 0.87. p < 0.0001. Figure 3B).

**Figure 4:**
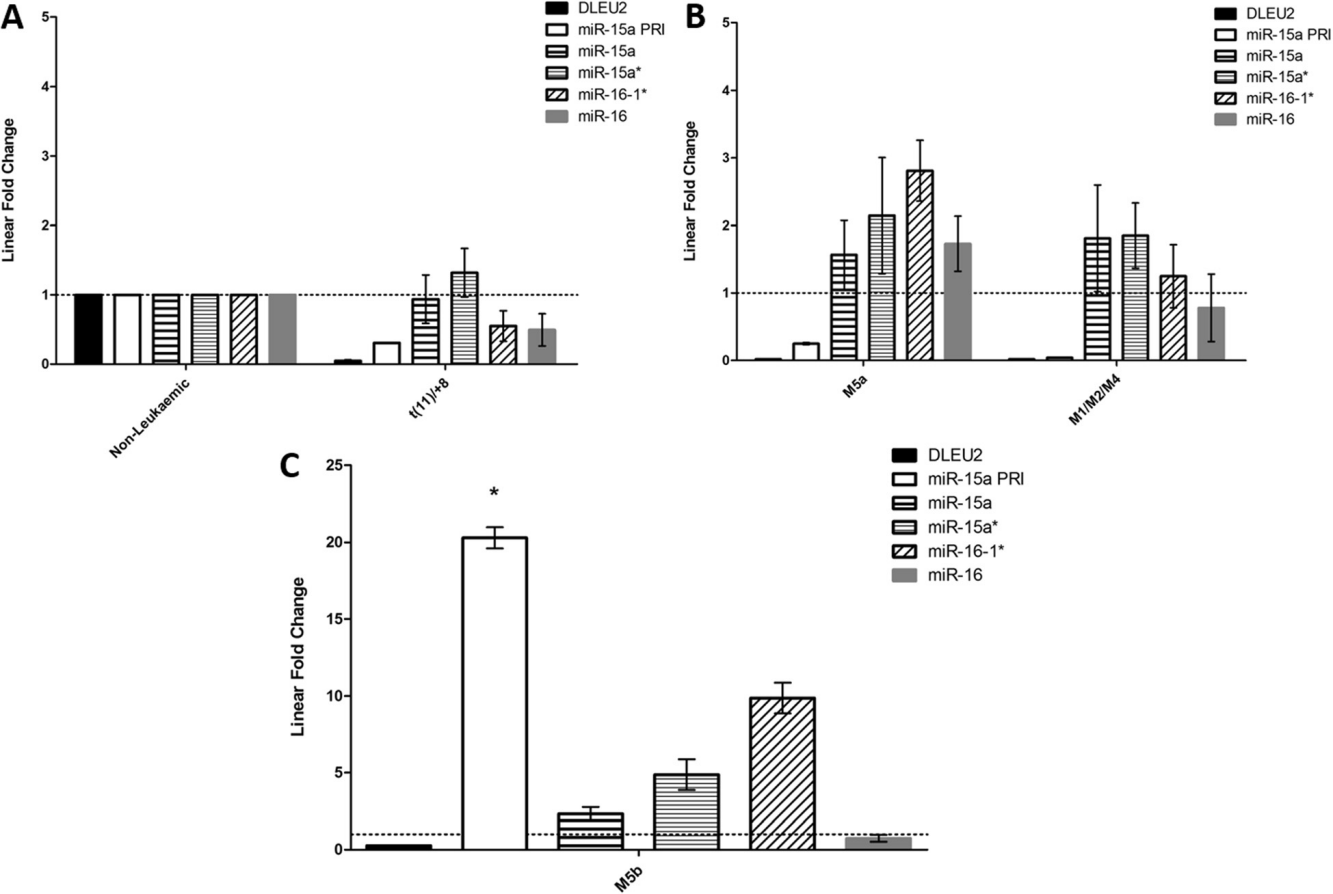
Gene, primary, miRNA and miRNA* expression for paediatric AML defined through clinical classification and *DLEU2 *Methylation subtyping. Mature microRNA expression, primary precursor transcript (PRI) and alternate miRNA isoform expression (*) from the *miR-15a/16-1* miRNA cluster embedded within *DLEU2* for paediatric AML patients (n = 26: 12 M5a, 5 M5b, 4 M1/M2/M4, 5 t(11)/+8 sub-group) all compared to non-leukaemic specimens (n = 30). Leukaemic groups refer to diagnostic bone marrow from paediatric patients. Non-leukaemic group consists of CD sorted cell populations (CD19+, CD33+, CD34+, CD45+)and patient remission specimens. Linear Fold Change (FC) is plotted using normalized data and the 2^-ΔΔCt^ method ± SD, and shows the fold change calculated from the means of each group. *DLEU2* gene expression is down-regulated in all subtypes. **A**. Fold change in expression comparing non-leukaemic to t(11)/+8 subtype. No significant differences in RNA expression are observed between non-leukaemic specimens and this subgroup. **B**. Fold change in expression comparing patients from subtype M5a to M1/M2/M4. DLEU2 is down-regulated in all subtypes (as previously described in Figure 2), and primary precursor for miR-15a also appears down-regulated (non-significant). M1/M2/M4 groupings do not show any mature miRNA expression changes (defined as >2-fold difference from non-leukaemic). Subtype M5a shows a 2.1-fold (±0.8 SD) increase in miR-15a* and a 2.81-fold (±0.4 SD) increase in miR-16-1*. **C**. FAB subtype M5b shows up-regulation of miR-15a PRI compared to non-leukaemic expression (20.29-fold (±0.6 SD) p < 0.001), with miR-15a* (4.87-fold ±1 SD) and miR-16-1* (9.86-fold ±1 SD) also up-regulated. M5b additionally shows a significant up-regulation of miR-15a PRI compared to t(11)/+8 sub-group samples (p < 0.05), and also from M5a and M1/M2/M4 sub-groups (p < 0.001).

Cases carrying 11q rearrangements are the most heterogeneous of paediatric AML [27,29,47], linked to 50–104 translocation fusion partners to date [29,46,48]. Trisomy 8 is a frequently reported aberration in adult and paediatric AML [29]. However little is known about the gain of chromosome 8 in isolation and its relationship to disease onset. As such this has been speculated to be a disease modulating secondary event [49]. Lower DNA methylation in the t(11)/+8 subgroup may potentially confer a better prognosis, as it is well documented that hypermethylation of the *DLEU2* region is involved in leukaemic transformation in adults [19,20,30]. Analysis of the t(11)/+8 subgroup in isolation also revealed no significant miRNA expression differences relative to non-leukaemic samples (Figure 4A), and it is well documented that *miR-15a/16-1* abnormalities are also associated with leukaemic transformation in adults [19,20]. We additionally identified a trend towards decreased risk of relapse in t(11)/+8 subgroup compared to other subtypes, and a trend towards better survival outcomes (Additional file 12). These analyses combined may elucidate a connection between *DLEU2* promoter DNA methylation (Figure 3B), miRNA expression (Figure 4A) and prognostic outcomes for this subgroup of paediatric AML (Additional file 12).

The classification of t(11)/+8 cases as an independent subgroup revealed a significant increase in pri-miR-15a expression in traditional FAB subtype M5b from non-leukaemic (20.29-fold, p < 0.001; Figure 4C) and from M5a/M1/M2/M4 (Figure 4B). The increase translates to a >2-fold increase in miR-15a, 4.87-fold increase in miR-15a*, and 9.86-fold increase in miR-16-1* expression. Based on *DLEU2* DNA methylation status, in conjunction with miRNA expression analysis, we speculate that pri-miR-15a expression alone may be a useful biomarker to distinguish M5b FAB subtype from all other AML subtypes.

## Conclusions

Previous research within paediatric AML has shown that well-defined cytogenetic subgroupings exhibit a wide range of genomic and epigenomic heterogeneity. Linking specific epigenetic features to clinical parameters has the potential to identify pathological drivers of disease and develop enhanced molecular approaches for diagnosis, prognosis and refinement of treatment. Profiling epigenetic regulators has provided clinically relevant biomarkers for adult cancers; however generally the same cannot be said of childhood cancers. Our analysis has identified hypermethylation induced down-regulation of the *DLEU2* gene in paediatric AML. The related expression changes of the embedded *miR-15a/16-1* microRNA cluster in paediatric AML has the potential to contribute to leukaemic transformation, with a switch from canonical to alternate miRNA family members, which in turn may modulate the expression of downstream regulatory genes. Treating paediatric AML model systems with epigenetic modifying drugs will allow a more comprehensive analysis towards the clinical applications and potential patient-focussed therapeutic interventions for children. Our results highlight the need for further specific interrogation of paediatric AML subtypes as distinctive biological entities, separate from adult disease. Further studies utilising larger patient cohorts are required to explore the complex interplay between the epigenetic regulation of genes harbouring microRNA, taking into account alternate miRNA transcript expression.

## Methods

### Samples

This study was approved by the Royal Children’s Hospital (RCH), Melbourne, Ethics Committee (HREC reference #27138E). Samples used consisted of snap frozen bone marrow specimens and archived bone marrow biopsies taken at diagnosis, patient remission/follow-up or at relapse from paediatric acute myeloid leukaemia (AML) cases. The diagnosis of AML was established according to the criteria of the French–American–British (FAB) classification by standard morphological and cytological methods. The evaluation for mutations in *MLL*, *FLT3, RUNX1* and *WT1* genes were assessed in a small number of patients as a part of the initial assessment. No patient recorded any chromosome 13 deletions or abnormalities listed in their clinical diagnoses. The median percentage of leukaemic blasts at patient diagnosis is 88% (67–93 95% CI).

Patient samples used in our study consisted of archived, air-dried bone marrow smear slides. The utility of these samples for DNA methylation and miRNA expression analysis has been outlined previously [34,50]. Cryogenically frozen patient bone marrows were also used where available. All patients were <18 years of age. We chose to focus specifically on the FAB subtype M5 (a and b) as it is a common subtype. Our cohort included comparable ratios between males and females, and also similar numbers of children that relapsed or not. (Additional file 5). Control samples from bone marrow of unrelated and unaffected children were analysed in parallel, as well as multiple cell lines, including adult leukaemia (REH [51], CCRF-CEM [52]), paediatric AML (Kasumi-1 [53], THP-1 [54], MV-4-11 and AML-193 [55]) obtained from American Type Culture Collection (ATCC) and subjected to characterisation using the ATCC Proficiency Standard program. Fluorescent Activated Cell Sorted (FACS) isolated haematopoietic progenitor cell populations from unrelated and unaffected paediatric donors (CD19+, CD33+, CD34+ and CD45+ populations, from herein known as CD sorted cells), were also used in this study. Our ‘non-leukaemic’ group consisted of all individual CD sorted cell populations as well as non-leukaemic remission and follow-up slides and bone marrow from paediatric patients. We compared the *DLEU2/Alt1* DNA methylation for all ‘non-leukaemic’ specimens, and found no significant differences across these non-leukaemic samples (Additional file 13).

### DNA extraction, quality control and methylation analysis

Genomic DNA was extracted using the phenol/chloroform method and bisulphite converted in accordance with manufacturer’s protocols, as reported previously [23]. DNA extracts were checked for quality and quantity using a NanoDrop® ND-1000 spectrophotometer (Thermo Fisher Scientific Inc., Scoresby, Victoria, Australia). All DNA was stored at -80°C.

Bisulphite conversion of genomic DNA was performed using the MethylEasy Xceed Bisulfite Modification Kit (Human Genetic Signatures, Sydney, AUST). The converted samples were processed by the Australian Genome Research Facility (AGRF, Melbourne, Australia) and analysed using the Illumina HumanMethylation450 (HM450) Bead Chip arrays according to manufacturer’s protocol. Illumina Genome Studio software was used to extract the raw M-values and probe intensities for downstream processing. Samples with a probe p-detection value of p <0.05 were retained. Data were quantile normalized using lumi [56] and analysed using LIMMA [57]. All analysis was performed using the R statistical software package on M-values that were converted to β-values (0 = unmethylated, 1 = fully methylated) for reporting and biological interpretation. To eliminate sex bias, probes hybridizing to sex chromosomes were filtered out, leaving 366,553 probes common to all samples in the final dataset. Further filtering was based on the degree of difference in β between sample groups, indicated as ∆β calculated using the formula:

$$\Delta \beta =\mathrm{mean}\beta \mathrm{value}\left(\mathrm{Leukaemic}\right)-\mathrm{mean}\beta \mathrm{value}\left(\mathrm{Non}-\mathrm{Leukaemic}\right)$$

SEQUENOM MassARRAY® EpiTYPER® was used to measure locus-specific methylation. Sixty-one samples were analysed, including the 42 analysed on the HM450 platform (Additional file 5), an additional 3 cell lines, 8 leukaemic and 8 non-leukaemic whole bone marrow aspirate samples. Primers for analysis were designed using SEQUENOM EpiDesigner software (http://www.epidesigner.com) and the sequences for these are listed in Additional file 14. Gene ontology and pathway analysis of genes associated with significantly altered DNA methylation probes were analysed through the use of IPA (Ingenuity® Systems, http://www.ingenuity.com) and GOrilla [58].

### RNA extraction, quality control and expression analysis

Small RNA extraction was performed using TRIzol® (Ambion®) for patient bone marrow samples, mononuclear cells and cell lines in accordance with the manufacturer’s instructions, or the High Pure miRNA Isolation Kit (Roche) for archived slide samples as previously described [34]. Before RNA extraction of fresh bone marrow aspirates, samples were processed using Ficoll-Paque™ (GE Healthcare, Piscataway USA) to isolate the mononuclear cell population. This was immediately cryo-frozen or stored in RNA*later*® (Ambion® by Life Technologies, Mulgrave, Victoria Australia) for later extraction. The concentration and purity of all RNA samples was assessed using the NanoDrop® ND-1000 spectrophotometer (Thermo Fisher Scientific Inc., Scorsby, Victoria, Australia). All RNA was stored at -80°C.

Seventy samples were used in the interrogation of miRNA expression, 64 of which were also used for HM450 and SEQUENOM methylation analysis (Additional file 5). TaqMan® MicroRNA Reverse Transcription kit and singleplex TaqMan ® microRNA Assays (Applied Biosystems, Life Technologies) (assays listed in Additional file 15) were utilised according to the manufacturer’s instructions before routine quantitative real-time PCR (qRT-PCR) was performed using Applied Biosystems 7300 Sequence Detection System. A subset of 29 high quality samples which included all cell lines as previously described, CD19+, CD33+, CD34+, CD45+ as well as 8 leukaemic and 9 non-leukaemic patient whole bone marrows, were used for gene and pri-miR expression analysis. This was due to longer RNA species often being degraded in archived specimens [59,60]. The SuperScript® VILO™ cDNA Synthesis Kit (Life Technologies) and TaqMan® gene expression assays were used for gene/pri-miR expression as per manufacturer’s instructions (Additional file 15 and Additional file 16) before performing qRT-PCR analysis according to the manufacturer’s instructions. All qRT-PCR samples were analysed in duplicate.

Expression data was normalized using the synthetic small nucleolar RNA, C/D Box 44 (RNU44) and glyceraldehyde-3-phosphate dehydrogenase (GAPDH) for gene/pri-miR expression; and RNU44 and hsa-miR-26b for miRNA analysis, previously identified as having the greatest stability in our samples [34]. Data were analysed using DataAssist™ software (v.3.0, Applied Biosystems, Life Technologies: http://www.lifetechnologies.com/au/en/home/technical-resources/software-downloads/dataassist-software), and all p-values were adjusted using Benjamini-Hochberg False Discovery Rate.

All analyses used the combined average of non-leukaemic primary bone marrow tissue and CD sorted non-leukaemic cell expression values as the Reference Group (calibrator). Fold Change (FC) was calculated using the Livak method of 2^-∆∆C^_t_[61] plotting fold change and associated p-values. The fold change reported here is the difference of the means of each group, and a fold change of >2 between disease and non-leukaemic groups were considered noteworthy. microRNA gene target prediction was assessed using microRNA.org [62,63] and miRWalk [64]. The top 500 target genes for each miRNA (Additional file 17) were used for gene ontology/pathway analysis (Additional file 10) by IPA (Ingenuity® Systems, http://www.ingenuity.com) and GOrilla [58].

### Statistical methods

Where groups were compared with more than one variable measured (non-repeated measures), a Two-Way ANOVA with Bonferroni Post-testing was performed. Where groups were compared with only one variable measured, a Kruskal-Wallis test was performed with Dunns Post-testing. These analyses were undertaken on normalized data. Correlation assessments utilised Spearman Rank tests. Two-tailed p-values were utilised for assessment of statistical significance, and results with p-values p < 0.05 were considered to be statistically significant. Survival and relapse curves were estimated by the Kaplan-Meier method. Statistical analyses were performed with GraphPad PRISM (Version 5) for Windows, (GraphPad Software, USA, http://www.graphpad.com). CI refers to 95% Confidence Intervals.

## Abbreviations

AML: Acute myeloid leukaemia; CN-AML: Cytogenetically normal acute myeloid leukaemia; DLEU2: Deleted in lymphocytic leukaemia 2; DMP: Differentially methylated probe; FAB: French-American-British leukaemia classification method; HM450: Illumina humanmethylation450 bead chip array; miR: microRNA/miRNA; Pri-miRNA: Primary microRNA; TSS: Transcriptional start site.

## Competing interests

The authors declare that they have no competing interests.

## Authors’ contributions

MH and MP-B carried out cell sorting, extractions and the accumulation of samples. JN assisted in accumulation of samples, extractions, conversions and assay running, as well as preliminary data analysis. FM and NG facilitated sample procurement and conceived the study. ZC participated in statistical and data analysis, study concepts and helped to draft the manuscript. RS and NW conceived the study, acquired the samples, interpreted the data, participated in drafting the manuscript and provided critical revisions for the approved final version. LM designed the study, acquired the samples, organised sample processing, participated in sample processing, undertook all data analysis and statistical evaluations and drafted the manuscript. All authors read and approved the final manuscript.

## Supplementary Material

Additional file 1 HM450 methylation heat map of the top 137 significantly differentially methylated probes between paediatric AML (FAB subtype M5) compared to non-leukaemic controls. Click here for file

Additional file 2 Paediatric AML DNA methylation results compared to non-leukaemic methylation for the differentially methylated probes identified using HM450. Click here for file

Additional file 3 DNA methylation cross-platform validation between HM450 and SEQUENOM MassARRAY® EpiTYPER®. Click here for file

Additional file 4 CpG locations captured by SEQUENOM MassARRAY® EpiTYPER®, including the HM450 probes. Click here for file

Additional file 5 Patient information for all paediatric AML patients used for HM450 DNA methylation interrogation, SEQUENOM MassARRAY® EpiTYPER® validation and gene expression analysis. Click here for file

Additional file 6 HM450 probe methylation values for the 13q4 region for paediatric AML (FAB M5) compared to non-leukaemic controls. Click here for file

Additional file 7 **Correlation between *DLEU2/Alt1* promoter DNA methylation and *DLEU2* gene expression.** Click here for file

Additional file 8 **HM450 probe methylation and expression dynamics of paediatric AML compared to non-leukaemic controls for the alternate miR-16 cluster, *miR-15b/16-2 *** **embedded in *SMC4 *** **on chromosome 3.** Click here for file

Additional file 9 Comparison of individual patient miRNA miR-15a/16-1 cluster expression. Click here for file

Additional file 10 Top Gene Ontology terms using GOrilla gene ontology identification tool. Click here for file

Additional file 11 ***DLEU2* promoter DNA methylation divided into paediatric AML patient clinical diagnostic observations.** Click here for file

Additional file 12 **Survival and relapse curves of paediatric AML, including a newly defined sub-group based on *DLEU2* promoter DNA methylation.** Click here for file

Additional file 13 ***DLEU2 *** **DNA methylation comparison of the three ‘non-leukaemic’ sample types.** Click here for file

Additional file 14 **Probes used in SEQUENOM MassARRAY® EpiTYPER® validation analysis of *DLEU2/Alt1 *** **HM450 promoter probes located on Chromosome 13.** Click here for file

Additional file 15 ***miR-15a/16-1 *** **cluster TaqMan® microRNA and primary precursor expression assays utilized, including Normalization references.** Click here for file

Additional file 16 TaqMan® gene expression assays utilized. Click here for file

Additional file 17 **The top 500 potential target genes identified for each differentially expressed *miR-15a/16-1 *** **cluster microRNA member in paediatric AML.** Click here for file
