# Supplementary material for: Hypermethylation and down-regulation of DLEU2 in paediatric acute myeloid leukaemia independent of embedded tumour suppressor miR-15a/16-1
Source: Mol Cancer. 2014 May 24;13:123. doi: 10.1186/1476-4598-13-123 (PMC4050407; doi:10.1186/1476-4598-13-123)
Supplement: Additional file 1 — HM450 methylation heat map of the top 137 significantly differentially methylated probes between paediatric AML (FAB subtype M5) compared to non-leukaemic controls. [file 1476-4598-13-123-S1.pdf]

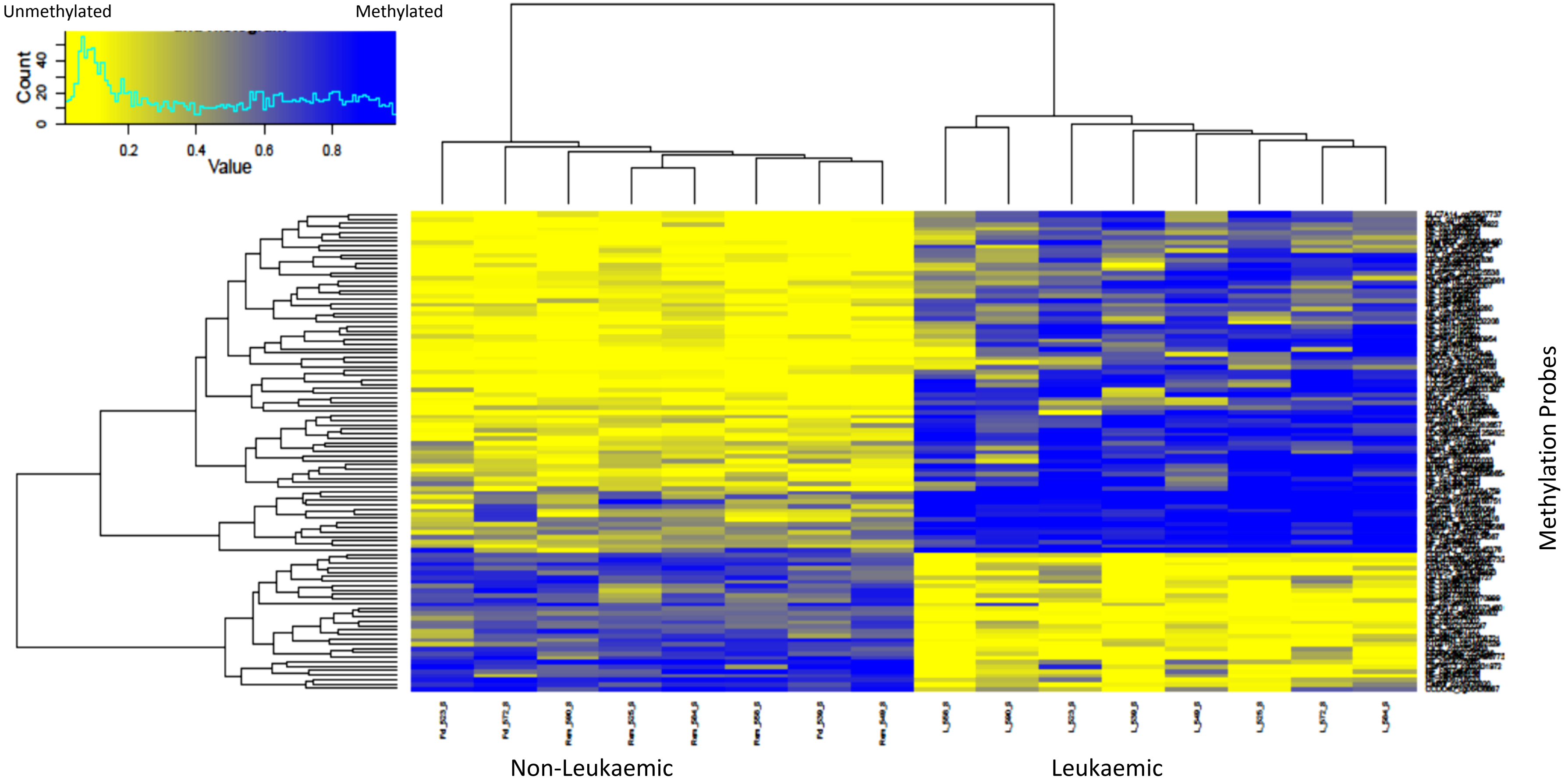


**Additional File 1: HM450 methylation heat map of the top 137 significantly differentially methylated probes between paediatric AML (FAB subtype M5) compared to non-leukaemic controls**

After quality control procedures and normalization/corrections of the HM450 raw data (outlined in Materials and Methods), 366,553 good quality probes were left for disease related differential methylation analysis. This heat map shows the most differentially methylated probes with an adjusted sample P-value <0.01 and a Δβ change of >0.4/<-0.4 totalling 137 probes. Unmethylated probes (0%) are represented in yellow, whilst fully methylated probes are in blue (100%). Leukaemic group refers to diagnostic bone marrow from paediatric patients. Non-Leukaemic group consists of CD sorted cell populations (CD19+, CD33+, CD34+, CD45+), normal cell line and patient remission specimens
