# Supplementary material for: Hypermethylation and down-regulation of DLEU2 in paediatric acute myeloid leukaemia independent of embedded tumour suppressor miR-15a/16-1
Source: Mol Cancer. 2014 May 24;13:123. doi: 10.1186/1476-4598-13-123 (PMC4050407; doi:10.1186/1476-4598-13-123)
Supplement: Additional file 2 — Paediatric AML DNA methylation results compared to non-leukaemic methylation for the differentially methylated probes identified using HM450. [file 1476-4598-13-123-S2.pdf]

Additional File 2: Paediatric AML DNA methylation results compared to non-leukaemic methylation for the differentially methylated probes identified using HM450

HM450 Probe	Leukaemic methylation	Non-leukaemic methylation	Difference	p-Value
CG10068417	53%	16%	37%	<0.001
CG09524946	36%	9%	27%	<0.001
CG07121900	62%	28%	34%	<0.001
CG05394800	62%	9%	53%	<0.001
CG20529344	63%	25%	38%	<0.001
CG12883980	61%	22%	39%	<0.001
CG23575603	88%	60%	28%	<0.001
**AVERAGE**	**60.7%**	**24.14%**	**36.6%**	
