# Supplementary material for: Hypermethylation and down-regulation of DLEU2 in paediatric acute myeloid leukaemia independent of embedded tumour suppressor miR-15a/16-1
Source: Mol Cancer. 2014 May 24;13:123. doi: 10.1186/1476-4598-13-123 (PMC4050407; doi:10.1186/1476-4598-13-123)
Supplement: Additional file 3 — DNA methylation cross-platform validation between HM450 and SEQUENOM MassARRAY® EpiTYPER®. [file 1476-4598-13-123-S3.pdf]

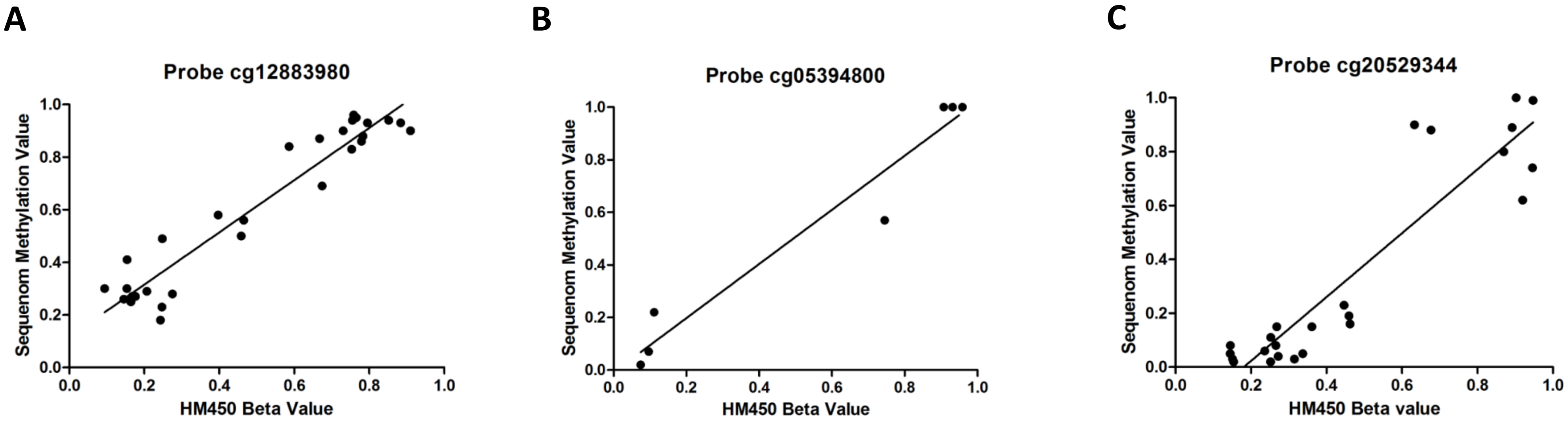

Additional File 3: DNA methylation cross-platform validation between HM450 and SEQUENOM MassARRAY® EpiTYPER®

Cross platform validation was undertaken to confirm the HM450 DNA methylation results we obtained using SEQUENOM MassARRAY® EpiTYPER® technology. The same paediatric AML and non-leukaemic samples were analysed using the two platforms, and correlations of resultant *DLEU2* probe DNA methylation results shown here. Strong correlations were obtained for all analyses

A) cg12883980: Spearman R=0.8535, P<0.0001

B) cg05394800: Spearman R=0.9636, P=0.0028. Due to the degraded nature of patient slide samples, cg053941800 contains fewer numbers of useable samples

C) cg20529344: Spearman R=0.8422, P<0.0001
