# Supplementary material for: Hypermethylation and down-regulation of DLEU2 in paediatric acute myeloid leukaemia independent of embedded tumour suppressor miR-15a/16-1
Source: Mol Cancer. 2014 May 24;13:123. doi: 10.1186/1476-4598-13-123 (PMC4050407; doi:10.1186/1476-4598-13-123)
Supplement: Additional file 4 — CpG locations captured by SEQUENOM MassARRAY® EpiTYPER®, including the HM450 probes. [file 1476-4598-13-123-S4.pdf]

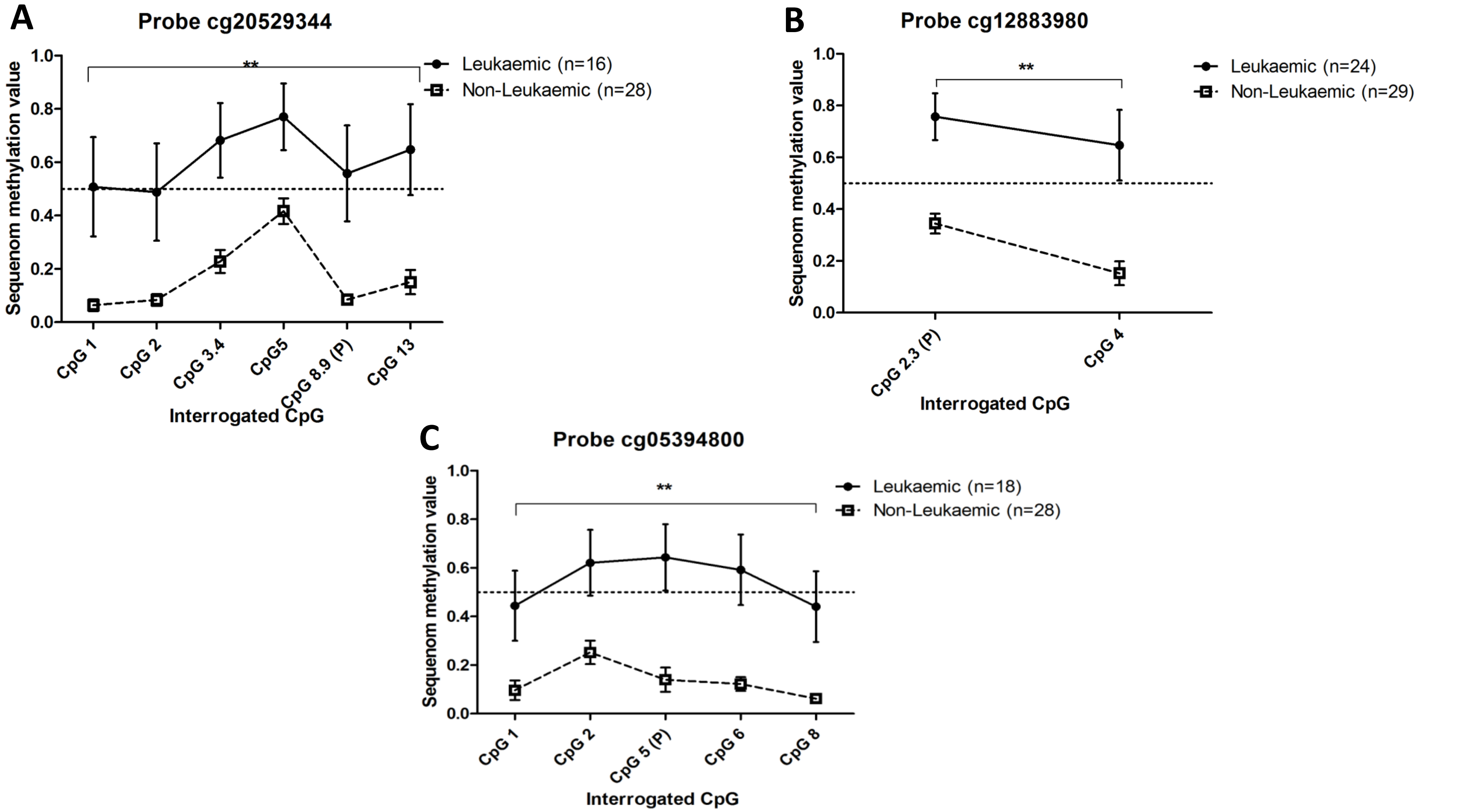

Additional File 4: CpG locations captured by SEQUENOM MassARRAY® EpiTYPER®, including the HM450 probes

Validation of HM450 probes for the *DLEU2/Alt1* promoter region using a larger cohort of paediatric AML and non-leukaemic specimens. Using SEQUENOM a number of additional CpG dinucleotides were investigated in addition to those captured in HM450 array. The HM450 probe location is denoted by (P), and methylation values range from 0.0 (0% Methylation) to 1.0 (100% methylation), to 95% CI. Leukaemic group refers to diagnostic bone marrow from paediatric patients. Non-Leukaemic group consists of CD sorted cell populations (CD19+, CD33+, CD34+, CD45+), normal cell line and patient remission specimens.

A) HM450 probe cg20529344: Four individual, and two clustered CpG dinucleotides were captured, including that interrogated by the HM450 probe. All were significantly differentially methylated between paediatric AML and non-leukaemic samples. Leukaemic vs Non-Leukaemic analysis P<0.001 for all CpGs

B) HM450 probe cg12883980: One individual and one clustered CpG dinucleotide were captured, including that interrogated by the HM450 probe. All were significantly differentially methylated between Paediatric AML and non-leukaemic samples. Leukaemic vs Non-Leukaemic analysis P<0.001 for all CpGs

C) HM450 probe cg5394800: Five individual CpG dinucleotides were captured by this assay, including that interrogated by the HM450. All were significantly differentially methylated between Paediatric AML and non-leukaemic samples. Leukaemic vs Non-Leukaemic analysis P<0.001 for all CpGs**.**
