# Supplementary material for: Hypermethylation and down-regulation of DLEU2 in paediatric acute myeloid leukaemia independent of embedded tumour suppressor miR-15a/16-1
Source: Mol Cancer. 2014 May 24;13:123. doi: 10.1186/1476-4598-13-123 (PMC4050407; doi:10.1186/1476-4598-13-123)
Supplement: Additional file 5 — Patient information for all paediatric AML patients used for HM450 DNA methylation interrogation, SEQUENOM MassARRAY® EpiTYPER® validation and gene expression analysis. [file 1476-4598-13-123-S5.pdf]

Additional File 5: Patient information for all paediatric AML patients used for HM450 DNA methylation interrogation, SEQUENOM MassARRAY® EpiTYPER® validation and gene expression analysis

Characteristic	450K Methylation Cohort (n=42)	Sequenom Methylation Cohort (n=61)	Quantitative RT-PCR (expression cohort) (n=70)
Samples used, no. (%)			
Primary Leukaemic Samples	18 (43)	24 (39)	27 (39)
Matched relapse	2 (5)	4 (7)	4 (6)
Matched non-Leukaemic	13 (31)	14 (23)	20 (28)
Non-Matched non-Leukaemic	4 (9)	11 (18)	11 (16)
Cell Lines	5 (12)	8 (13)	8 (11)
Age at diagnosis			
Mean	7.5	9.98	10.33
Range	0.54-15.49	0.54-17.28	0.54-17.28
Sex, no. (%)			
Female	11 (61)	14 (58)	15 (56)
Male	7 (39)	10 (42)	12 (44)
FAB Classifications, no. (%)			
M5a	13 (72)	14 (58)	16 (59)
M5b	5 (28)	6 (25)	7 (26)
Other		4 (17)	4 (15)
Genetic Abnormalities, no. (%)			
WT1		1 (4)	1 (4)
RUNX1		2 (8)	2 (7)
MLL rearrangement	6 (33)	7 (28)	8 (29)
FLT-ITD			1 (4)
Other		1 (4)	1 (4)
Not Defined/None	12 (66)	14 (56)	14 (52)
Cytogenetics, no. (%)			
Normal Karyotype (CN-AML)	5 (28)	5 (21)	5 (18)
Hyperdiploid	4 (22)	4 (17)	6 (21)
Pseudodiploid		1 (4)	1 (4)
Complex Karyotype		1 (4)	1 (4)
Chr 11 rearrangement	6 (33)	8 (33)	8 (28)
+8	1 (6)	1 (4)	3 (11)
Other	2 (11)	4 (17)	4 (14)
Relapse Status, no. (%)			
No Relapse	11 (61)	11 (46)	12 (44)
Relapse/deceased	7 (39)	9 (38)	11 (41)
Not Recorded		4 (16)	4 (15)
Overlapping samples in analysis			
HM450 - SEQUENOM		42	
HM450 - qRT-PCR			42
SEQUENOM - qRT-PCR			61
Sample types			
Patient Bone Marrow	1	17	17
Patient Smear Film	33	31	41
