# Supplementary material for: Hypermethylation and down-regulation of DLEU2 in paediatric acute myeloid leukaemia independent of embedded tumour suppressor miR-15a/16-1
Source: Mol Cancer. 2014 May 24;13:123. doi: 10.1186/1476-4598-13-123 (PMC4050407; doi:10.1186/1476-4598-13-123)
Supplement: Additional file 6 — HM450 probe methylation values for the 13q4 region for paediatric AML (FAB M5) compared to non-leukaemic controls. [file 1476-4598-13-123-S6.pdf]

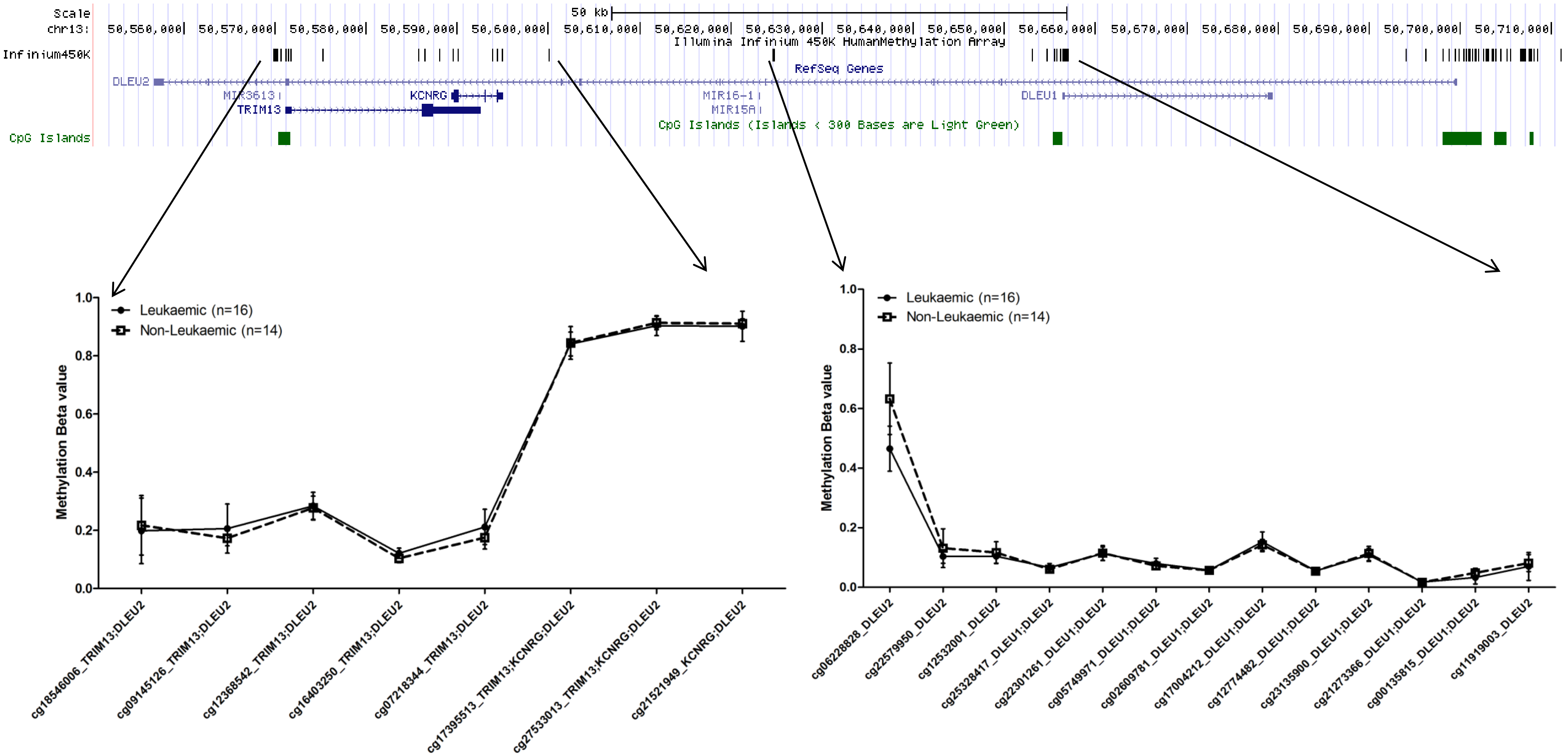

Additional File 6: HM450 probe methylation values for the 13q4 region for paediatric AML (FAB M5) compared to non-leukaemic controls

The methylation profile of paediatric AML patients compared to non-leukaemic signals was compared for all HM450 probes in chromosome 13q4 region, outside of the *DLEU2/Alt1* promoter region. The interrogation here included *DLEU1* promoter regions, *miR-15a/16-1* cluster, *TRIM13* and *KCNRG*. No differential methylation was recorded for any of the 13q4 region (95% CI). Leukaemic group refers to diagnostic bone marrow from paediatric patients. Non-Leukaemic group consists of CD sorted cell populations (CD19+, CD33+, CD34+, CD45+), normal cell line and patient remission specimens.
