# Supplementary material for: Hypermethylation and down-regulation of DLEU2 in paediatric acute myeloid leukaemia independent of embedded tumour suppressor miR-15a/16-1
Source: Mol Cancer. 2014 May 24;13:123. doi: 10.1186/1476-4598-13-123 (PMC4050407; doi:10.1186/1476-4598-13-123)
Supplement: Additional file 7 — Correlation between DLEU2/Alt1 promoter DNA methylation and DLEU2 gene expression. [file 1476-4598-13-123-S7.pdf]

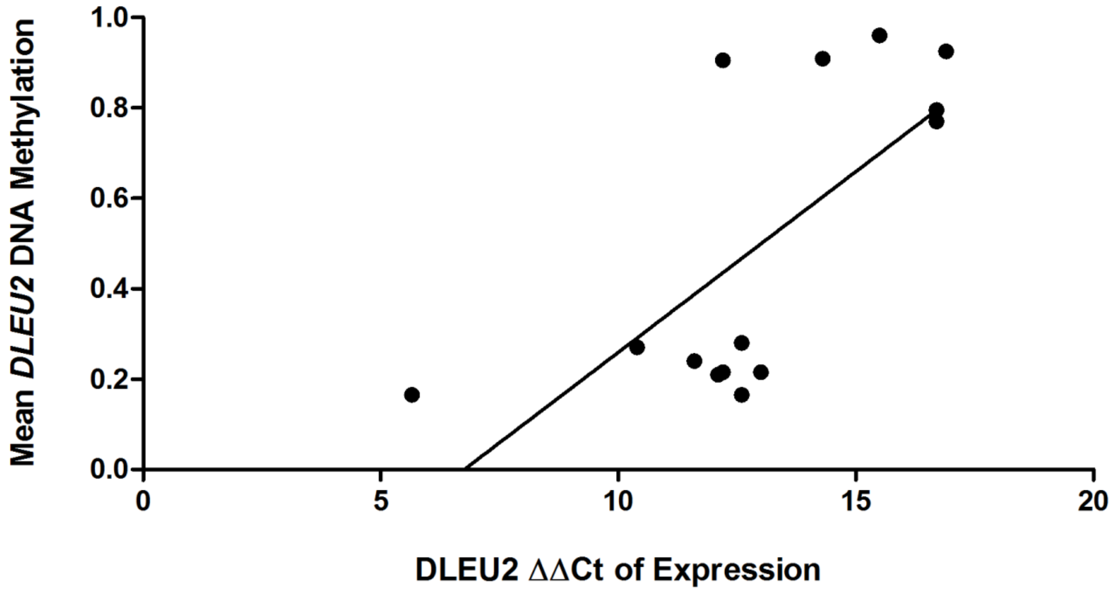

Additional File 7: Correlation between *DLEU2/Alt1* promoter DNA methylation and *DLEU2* gene expression

Methylation of the *DLEU2* region under investigation was compared to the DLEU2 gene expression values obtained after qRT-PCR analysis. The methylation values are represented from 0 (0.0) to 100% (1.0), where the methylation values obtained for all three HM450 probes have been combined. The expression values are shown in normalized $\Delta\Delta C_t$.Gene promoter DNA methylation and gene expression are significantly correlated, n=14 matched patient pairs, patient samples only, R^2=0.437, p=0.0001
