# Supplementary material for: Hypermethylation and down-regulation of DLEU2 in paediatric acute myeloid leukaemia independent of embedded tumour suppressor miR-15a/16-1
Source: Mol Cancer. 2014 May 24;13:123. doi: 10.1186/1476-4598-13-123 (PMC4050407; doi:10.1186/1476-4598-13-123)
Supplement: Additional file 8 — HM450 probe methylation and expression dynamics of paediatric AML compared to non-leukaemic controls for the alternate miR-16 cluster, miR-15b/16-2 embedded in SMC4 on chromosome 3. [file 1476-4598-13-123-S8.pdf]

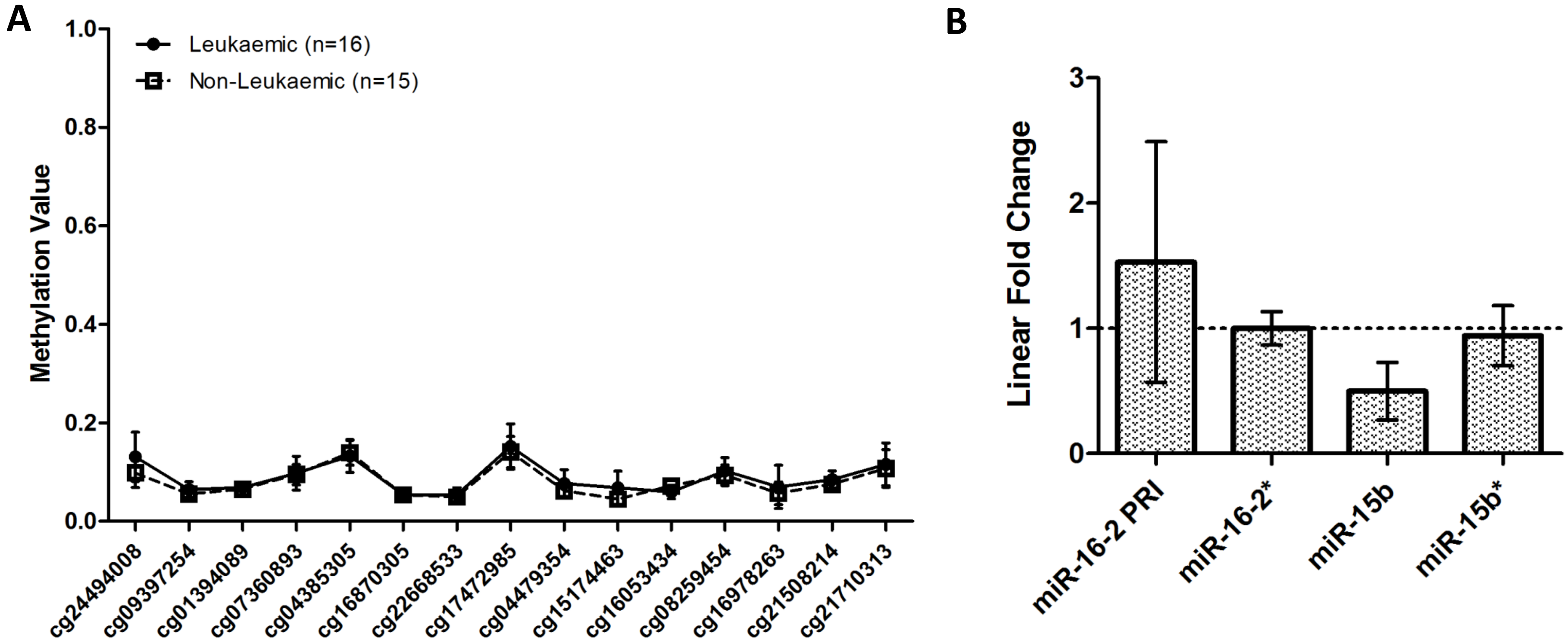

Additional File 8: HM450 probe methylation and expression dynamics of paediatric AML compared to non-leukaemic controls for the alternate miR-16 cluster, *miR-15b/16-2* embedded in *SMC4* on chromosome 3

The miR-16 mature microRNA is encoded for by two genes, the first on Chromosome 13 and embedded in *DLEU2*. The second gene sits within *SMC4* and is referred to as *miR-15b/16-2*.

A) There is no identifiable methylation difference between Paediatric AML HM450 DNA methylation results as compared to non-leukaemic samples using this platform (95% CI). Leukaemic group refers to diagnostic bone marrow from paediatric patients. Non-Leukaemic group consists of CD sorted cell populations (CD19+, CD33+, CD34+, CD45+), normal cell line and patient remission specimens.

B) Mature microRNA expression, including primary precursor transcript (PRI) and alternate miRNA expression (*), from the *miR-15b/16-2* miRNA cluster for paediatric AML (n=28, including the 10 used in Figure 2A) compared to non-leukaemic specimens represented by the dashed line-of-no-change (n=30, including the 13 used in Figure 2A). Here miR15b/16-2 PRI transcript is moderately, but not significantly, increased in paediatric AML compared to non-leukaemic expression (1.53 FC). This increase does not contribute to an increase in mature miR-16-2* (0.99 FC), miR-15b (0.5 FC) nor miR-15b* (0.94 FC) which show no change in expression in AML compared to non-leukaemic.
