# Supplementary material for: Hypermethylation and down-regulation of DLEU2 in paediatric acute myeloid leukaemia independent of embedded tumour suppressor miR-15a/16-1
Source: Mol Cancer. 2014 May 24;13:123. doi: 10.1186/1476-4598-13-123 (PMC4050407; doi:10.1186/1476-4598-13-123)
Supplement: Additional file 9 — Comparison of individual patient miRNA miR-15a/16-1 cluster expression. [file 1476-4598-13-123-S9.pdf]

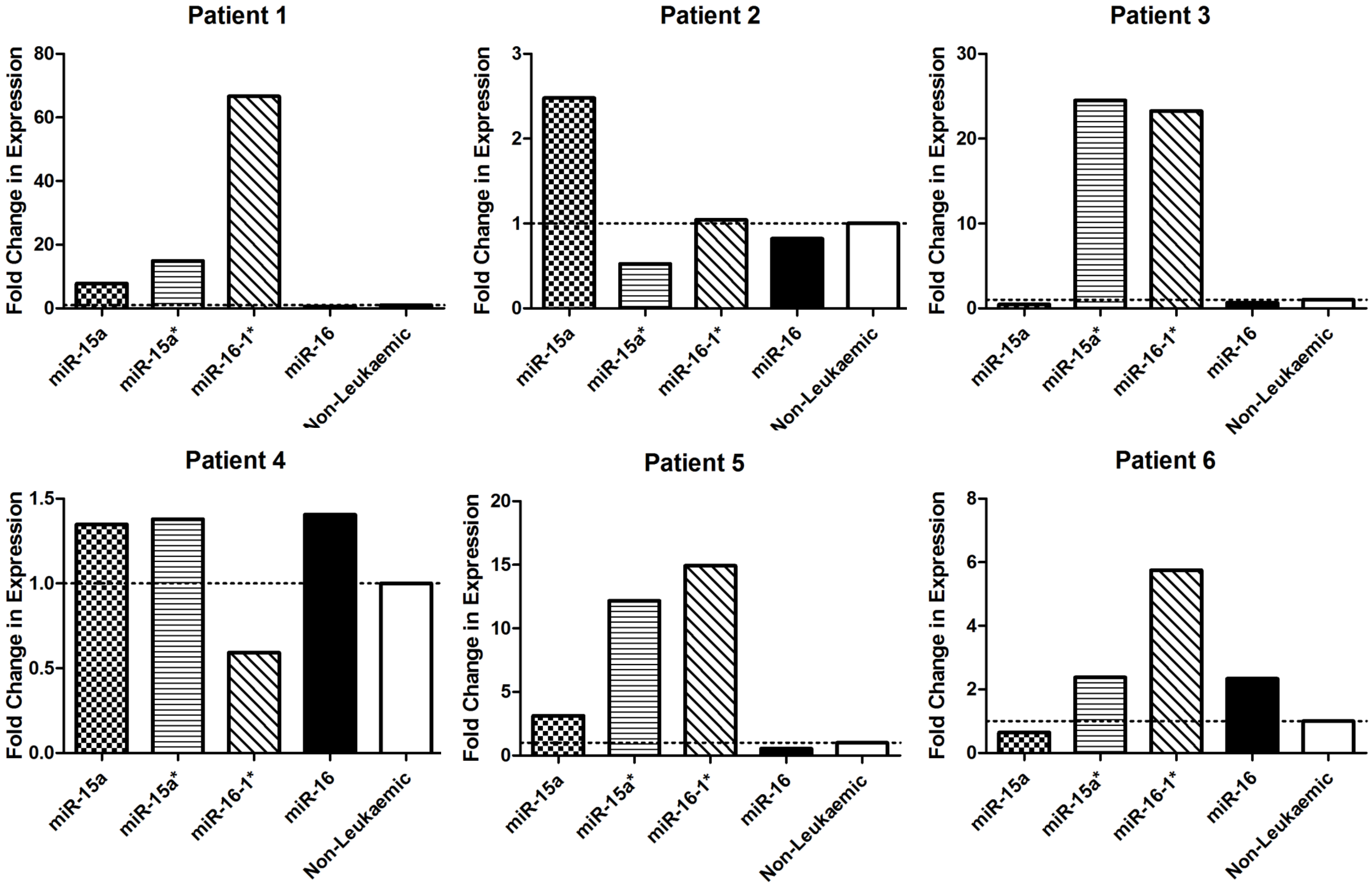

Additional File 9: Comparison of individual patient miRNA miR-15a/16-1 cluster expression

Examples of the individual patient leukaemic miRNA expression exhibited as linear fold change, normalized to miR-26b and RNU44, and compared to non-leukaemic expression. These values contributed to Figure 2, therefore elucidating some of the error bars for the AML cohort as a whole.
