# Supplementary material for: Hypermethylation and down-regulation of DLEU2 in paediatric acute myeloid leukaemia independent of embedded tumour suppressor miR-15a/16-1
Source: Mol Cancer. 2014 May 24;13:123. doi: 10.1186/1476-4598-13-123 (PMC4050407; doi:10.1186/1476-4598-13-123)
Supplement: Additional file 10 — Top Gene Ontology terms using GOrilla gene ontology identification tool. [file 1476-4598-13-123-S10.pdf]

Additional File 10: Top Gene Ontology terms using GOrilla gene ontology identification tool
The top 500 target genes for each differentially expressed *miR-15a/16-1* microRNA cluster member in paediatric AML were used for GOrilla gene ontology pathway analysis

miRNA	GO Term	Description	P-Value	Enrichment	Gene Example
**hsa-miR-16-1***	GO:0006396	RNA processing	7.95E-07	3.48	EMG1, RPP30, HNRNPR, CHERP, SRRM1
	GO:0000375	RNA splicing, via transesterification reactions	1.98E-05	4.57	SMNDC1, TXNL4A, HNRNPA0, SRSF10
	GO:0008380	RNA splicing	5.02E-05	3.81	PPARGC1A, NUDT21, MBNL2
	GO:0006397	mRNA processing	5.12E-05	3.43	CELF2, APOBEC2, SLU7
	GO:0000377	RNA splicing, via transesterification reactions with bulged adenosine as nucleophile	7.64E-05	4.57	SRRM1, NUDT21, TXNL4A
	GO:0000398	mRNA splicing, via spliceosome	7.64E-05	4.57	SLU7, HNRNPR, HNRNPA0
	GO:2001244	positive regulation of intrinsic apoptotic signaling pathway	4.29E-04	63.29	BCL2L11, DNM1L
	GO:2001242	regulation of intrinsic apoptotic signaling pathway	4.29E-04	63.29	BCL2L11, DNM1L
**hsa-miR-15a**	GO:0072384	organelle transport along microtubule	2.50E-04	23.52	DYNC1I1, MAP2K1, KIF1B
	GO:0007017	microtubule-based process	3.37E-04	10.45	EYA1, DYNC1I1, MAP7
	GO:0035239	tube morphogenesis	6.73E-04	17.33	LRP6, EYA1, DLL1
	GO:0010970	microtubule-based transport	7.09E-04	18.81	DYNC1I1, MAP2K1, KIF1B
	GO:0030705	cytoskeleton-dependent intracellular transport	7.09E-04	18.81	DYNC1I1, MAP2K1, KIF1B
	GO:0051656	establishment of organelle localization	7.09E-04	18.81	EYA1, MAP2K1, KIF1B
	GO:0030155	regulation of cell adhesion	8.86E-04	10.98	ARL2, SMAD7, MAP2K1, DLL1
**hsa-miR-15a***	GO:2001242	regulation of intrinsic apoptotic signaling pathway	1.92E-04	91.4	BCL2L11, PPIF
	GO:0090200	positive regulation of release of cytochrome c from mitochondria	1.92E-04	91.4	BCL2L11, PPIF
	GO:0010822	positive regulation of mitochondrion organization	4.46E-04	60.93	BCL2L11, PPIF
	GO:0090199	regulation of release of cytochrome c from mitochondria	4.46E-04	60.93	BCL2L11, PPIF
