# Supplementary material for: Hypermethylation and down-regulation of DLEU2 in paediatric acute myeloid leukaemia independent of embedded tumour suppressor miR-15a/16-1
Source: Mol Cancer. 2014 May 24;13:123. doi: 10.1186/1476-4598-13-123 (PMC4050407; doi:10.1186/1476-4598-13-123)
Supplement: Additional file 11 — DLEU2 promoter DNA methylation divided into paediatric AML patient clinical diagnostic observations. [file 1476-4598-13-123-S11.pdf]

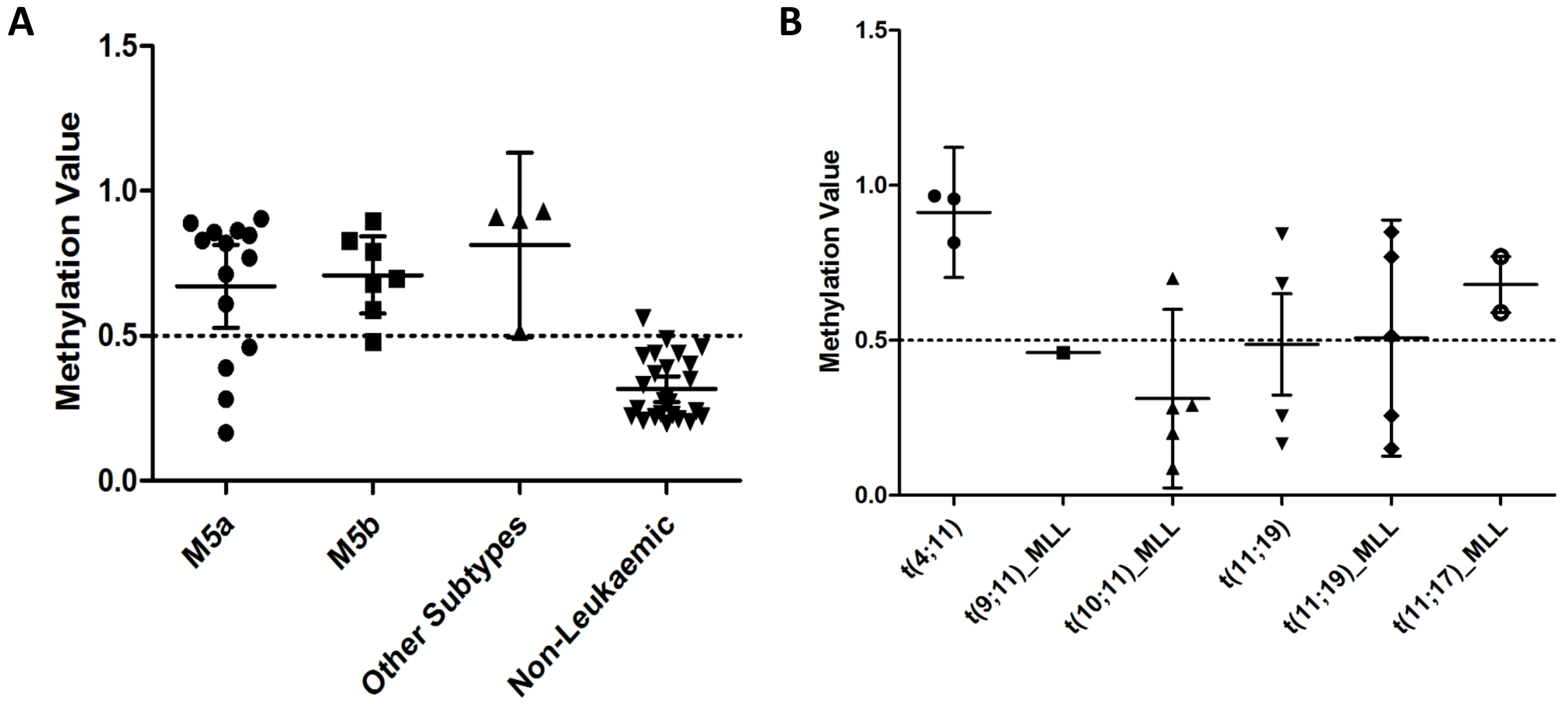

Additional File 11: *DLEU2* promoter DNA methylation divided into paediatric AML patient clinical diagnostic observations

DLEU2 promoter DNA methylation was investigated for paediatric AML patients in relation to their reported clinical diagnosis. DNA Methylation was measured from 0% (0.0) to 100% (1.0) methylation, to 95% CI. Non-Leukaemic group consists of CD sorted cell populations (CD19+, CD33+, CD34+, CD45+), normal cell line and patient remission specimens.

A) Paediatric AML methylation status of the *DLEU2* promoter region subdivided by clinical FAB identification and compared to non-leukaemic specimens, including those with known gene/chromosomal abnormalities and CN-AML cases. 'Other' subtype classification includes children diagnosed with M1, M2 and M4 AML. The DNA methylation values are depicted here for probe cg12883980, as an indication of the methylation at the *DLEU2* promoter region. A significant increase in DNA methylation was observed in M5a, M5b and 'Other' AML compared to non-leukaemic signatures ($P<0.05$). However *DLEU2* promoter DNA methylation at this level was unable to differentiate between M5a (mean methylation (MM)=0.67), M5b (MM=0.71) and Other subtypes (MM=0.81).

B) Further interrogation of DNA methylation of patients with recorded chromosome 11 abnormalities alone, including known gene translocations, eg. *MLL* as shown here. We utilized DNA methylation values from all three HM450 probes (cg05394800, cg20529344 and cg12883980). Certain AML patients with t(10;11), t(11;19) and t(9;11) regardless of known gene abnormalities (eg. *MLL*) show consistently reduced *DLEU2* promoter DNA methylation values.
