# Supplementary material for: Hypermethylation and down-regulation of DLEU2 in paediatric acute myeloid leukaemia independent of embedded tumour suppressor miR-15a/16-1
Source: Mol Cancer. 2014 May 24;13:123. doi: 10.1186/1476-4598-13-123 (PMC4050407; doi:10.1186/1476-4598-13-123)
Supplement: Additional file 12 — Survival and relapse curves of paediatric AML, including a newly defined sub-group based on DLEU2 promoter DNA methylation. [file 1476-4598-13-123-S12.pdf]

A

B

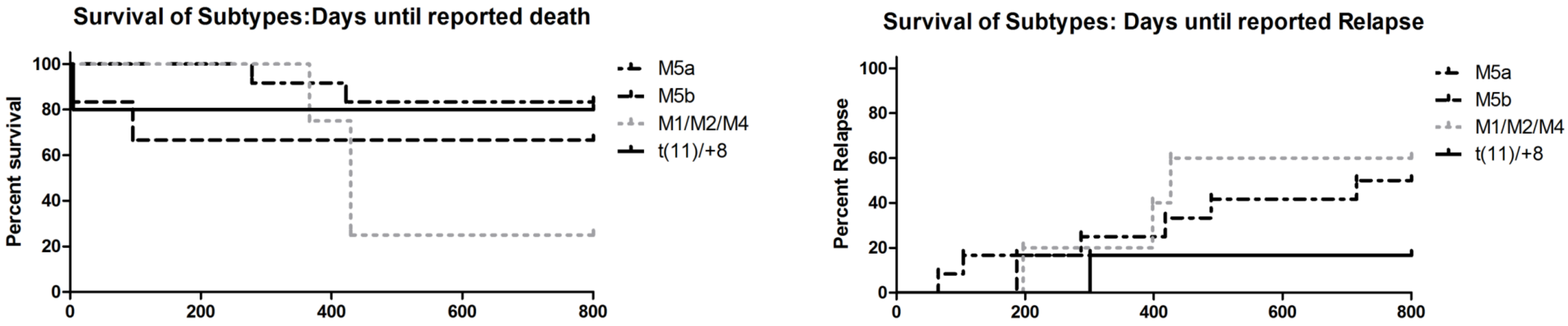

Additional File 12: Survival and relapse curves of paediatric AML, including a newly defined sub-group based on *DLEU2* promoter DNA methylation

Survival (A) and Relapse (B) curves of Paediatric AML, comparing FAB subtypes M5a, M5b, M1/M2/M4 and our newly defined 't(11)/+8 sub-group' based on the promoter DNA methylation of *DLEU2/Alt1*. Patient outcome was followed until 800 days (2.2 years) after initial diagnosis. Whilst patient numbers are low (M5a: n=12. M5b: n=6. M1/M2/M4: n=5. t(11)/+8: n=6) these data suggest based on *DLEU2/Alt1* DNA methylation may be a trend towards differential outcomes of paediatric patients.

A) Survival curves representing the number of patients who passed away during the study period. FAB subtype M5a has the highest overall survival rate (83.3% survival after 800 days), followed by t(11)/+8 (80%). M5b has intermediate survival rate (66.6%), and M1/M2/M4 the lowest survival for this cohort.

B) An additional measure of patient outcome in paediatric patients is the occurrence of disease bone marrow relapse during the study period. M1/M2/M4 subgrouping has the highest relapse potential (60%), followed by M5a (50%). M5b and t(11)/+8 sub-group are equally low in relapse status (16.6%).
