# Supplementary material for: Hypermethylation and down-regulation of DLEU2 in paediatric acute myeloid leukaemia independent of embedded tumour suppressor miR-15a/16-1
Source: Mol Cancer. 2014 May 24;13:123. doi: 10.1186/1476-4598-13-123 (PMC4050407; doi:10.1186/1476-4598-13-123)
Supplement: Additional file 13 — DLEU2 DNA methylation comparison of the three ‘non-leukaemic’ sample types. [file 1476-4598-13-123-S13.pdf]

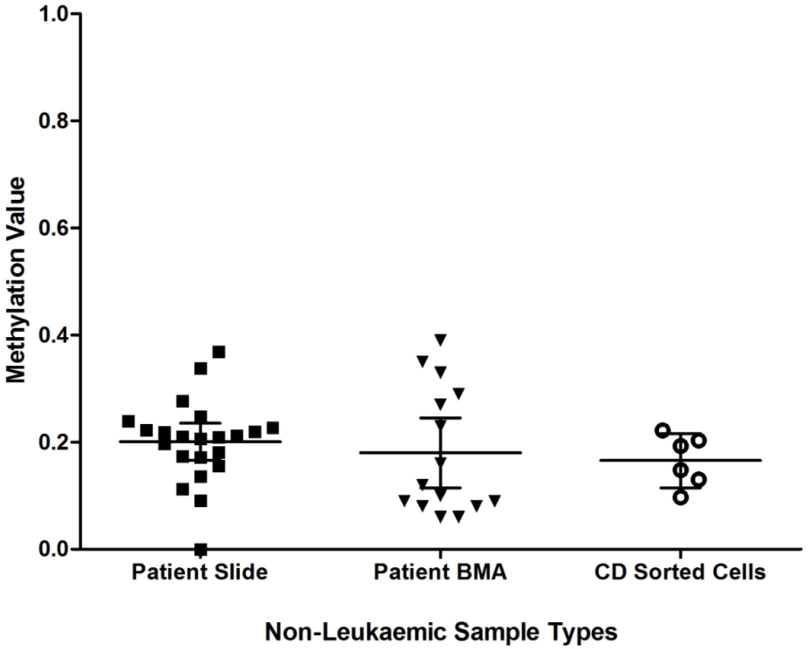

Additional File 13: *DLEU2* DNA methylation comparison of the three 'non-leukaemic' sample types

The DNA methylation of *DLEU2* promoter HM450 probes was compared for the potential 'non-leukaemic' samples identified for use in this study. Here we utilized DNA methylation values from all three HM450 probes (cg05394800 , cg20529344 and cg12883980), and DNA Methylation was measured from 0% (0.0) to 100% (1.0) methylation, to 95% CI. Patient Slide values refer to the non-leukaemic archived bone marrow aspirates obtained during this study (Mean Methylation (MM): 0.2008), which may be from Remission or Follow-up after treatment for Leukaemia. Patient BMA are snap-frozen patient non-leukaemic whole bone marrow aspirates (MM: 0.18)), which may be from Remission or Follow-up after treatment for Leukaemia. CD sorted cells refers to sorted non-affected non-leukaemic control donor whole bone marrow, FACS sorted into CD19+, CD33+, CD34+ and CD45+ compartments. The methylation values obtained from all CD sorted cells are combined to form the 'CD Sorted Cells' group (MM: 0.17). All DNA values from all groups was not significantly different, therefore we combined all values from the three groups to form the 'non-leukaemic' category in other analyses.
