# Supplementary material for: Hypermethylation and down-regulation of DLEU2 in paediatric acute myeloid leukaemia independent of embedded tumour suppressor miR-15a/16-1
Source: Mol Cancer. 2014 May 24;13:123. doi: 10.1186/1476-4598-13-123 (PMC4050407; doi:10.1186/1476-4598-13-123)
Supplement: Additional file 14 — Probes used in SEQUENOM MassARRAY® EpiTYPER® validation analysis of DLEU2/Alt1 HM450 promoter probes located on Chromosome 13. [file 1476-4598-13-123-S14.pdf]

Additional File 14: Probes used in SEQUENOM MassARRAY® EpiTYPER® validation analysis of *DLEU2/Alt1* HM450 promoter probes located on Chromosome 13

HM450 Probe	Probe Location	Validation Left Primer	Validation Right Primer
CG05394800	chr13:50707051-50707051	TTTGTTTATGAAAATTTGGGGTATT	CACCAAAAACTTACAAACCACTAAAA
CG20529344	chr13:50707428-50707428	GGAGTTGTTTAGAAAAGATGGGTTT	AAAACCCTTAAAATTATTCCCTAACC
CG12883980	chr13:50707587-50707587	GGGATATTAGAGGATTTAGGGAGT	AATCTTAAAATAAACCCCCAACCT
