# Supplementary material for: Hypermethylation and down-regulation of DLEU2 in paediatric acute myeloid leukaemia independent of embedded tumour suppressor miR-15a/16-1
Source: Mol Cancer. 2014 May 24;13:123. doi: 10.1186/1476-4598-13-123 (PMC4050407; doi:10.1186/1476-4598-13-123)
Supplement: Additional file 15 — miR-15a/16-1 cluster TaqMan® microRNA and primary precursor expression assays utilized, including Normalization references. [file 1476-4598-13-123-S15.pdf]

Additional File 15: *miR-15a/16-1* cluster TaqMan® microRNA and primary precursor expression assays utilized, including Normalization references

miRNA Name	Species	Sequence	Location	miRBase Accession no.	Type
miR-16	Human	UAGCAGCACGUAAAUAUUGGCG	Chr.13: 50623109 - 50623197 [-]	MI0000070	Target
miR-16-1*	Human	CCAGUAUUAACUGUGCUGCUGA	Chr.13: 50623109 - 50623197 [-]	MI0000070	Target
miR-15a	Human	UAGCAGCACAUAAUGGUUUGUG	Chr.13: 50623255 - 50623337 [-]	MI0000069	Target
miR-15a*	Human	CAGGCCAUAUUGUGCUGCCUCA	Chr.13: 50623255 - 50623337 [-]	MI0000069	Target
miR-16-2*	Human	CCAAUAUUACUGUGCUGCUUUA	Chr.3: 160122533 - 160122613 [+]	MI0000115	Target
miR-15b	Human	UAGCAGCACAUCAUGGUUUACA	Chr.3: 160122376 - 160122473 [+]	MI0000438	Target
miR-15b*	Human	CGAAUCAUUAUUUGCUGCUCUA	Chr.3: 160122376 - 160122473 [+]	MI0000438	Target
miR-26b	Human	UUCAAGUAAUUCAGGAUAGGU	Chr.2: 219267369 - 219267445 [+]	MI0000084	Reference
RNU44	Human	CCTGGATGATGATAGCAAATGCTGACTGAACATGAAGGTCTTAATTAGCTCTAACTGACT	Synthetic RNA construct		Reference
PRI-miR-15a	Human	CCUUGGAGUAAAGUAGCAGCACAUAAUGGUUUGUGGAUUUUGAAAAGGUGCAGGCCAUAUUGUGCUGCCUCAAAAAUACAAGG	Chr. 13: 50623255-50623337 [-]	MI0000069	Stem-loop Target
PRI-miR-16-2	Human	GUUCCACUCUAGCAGCACGUAAAUAUUGGCGUAGUGAAAUAUAUAUUAAACACCAAUAUUACUGUGCUGCUUUAGUGUGAC	Chr.3: 160122533-160122613 [+]	MI0000115	Stem-loop Target
