# Supplementary material for: Hypermethylation and down-regulation of DLEU2 in paediatric acute myeloid leukaemia independent of embedded tumour suppressor miR-15a/16-1
Source: Mol Cancer. 2014 May 24;13:123. doi: 10.1186/1476-4598-13-123 (PMC4050407; doi:10.1186/1476-4598-13-123)
Supplement: Additional file 16 — TaqMan® gene expression assays utilized. [file 1476-4598-13-123-S16.pdf]

Additional File 16: TaqMan® gene expression assays utilized

Gene	Species	Location	RefSeq ID	Exon Boundry	Assay Location	Assay ID
DLEU1	Human	Chr. 13: 50656414-50679433	NR_002605.1	2	445	Hs00705554_s1
DLEU2	Human	Chr. 13: 50556688-50699677	NR_002612.1	6-7	811	Hs00863925_m1
TRIM13	Human	Chr. 13: 50571143-50592603	NM_001007278.1/005798.3/052811.2/213590.1	4/3/2	5140/4941/5026/5476	H200328634_s1
